# Machine learning with taxonomic family delimitation aids in the classification of ephemeral beaked whale events in passive acoustic monitoring

**DOI:** 10.1371/journal.pone.0304744

**Published:** 2024-06-04

**Authors:** Alba Solsona-Berga, Annamaria I. DeAngelis, Danielle M. Cholewiak, Jennifer S. Trickey, Liam Mueller-Brennan, Kaitlin E. Frasier, Sofie M. Van Parijs, Simone Baumann-Pickering

**Affiliations:** 1 Scripps Institution of Oceanography, University of California San Diego, La Jolla, California, United States of America; 2 Northeast Fisheries Science Center, National Marine Fisheries Service, National Oceanic and Atmospheric Administration, Woods Hole, Massachusetts, United States of America; Naturhistoriska riksmuseet, SWEDEN

## Abstract

Passive acoustic monitoring is an essential tool for studying beaked whale populations. This approach can monitor elusive and pelagic species, but the volume of data it generates has overwhelmed researchers’ ability to quantify species occurrence for effective conservation and management efforts. Automation of data processing is crucial, and machine learning algorithms can rapidly identify species using their sounds. Beaked whale acoustic events, often infrequent and ephemeral, can be missed when co-occurring with signals of more abundant, and acoustically active species that dominate acoustic recordings. Prior efforts on large-scale classification of beaked whale signals with deep neural networks (DNNs) have approached the class as one of many classes, including other odontocete species and anthropogenic signals. That approach tends to miss ephemeral events in favor of more common and dominant classes. Here, we describe a DNN method for improved classification of beaked whale species using an extensive dataset from the western North Atlantic. We demonstrate that by training a DNN to focus on the taxonomic family of beaked whales, ephemeral events were correctly and efficiently identified to species, even with few echolocation clicks. By retrieving ephemeral events, this method can support improved estimation of beaked whale occurrence in regions of high odontocete acoustic activity.

## Introduction

Monitoring species is a tremendous and fundamentally important effort to answering many theoretical questions in ecology, and establishing species management and conservation strategies. The fact that large marine species such as beaked whales (family Ziphiidae) are still being discovered in the 21^st^ century [1–3] demonstrates the difficulty of monitoring and studying megafauna in pelagic habitats. There are now 24 identified species of beaked whales [4], all of which inhabit deep waters and are notoriously difficult to sight. One-third of these species are *Data Deficient* on the International Union for Conservation of Nature (IUCN) Red List of Threatened Species, meaning there is insufficient information to assess risk of extinction, and half have been recently listed as *Least Concern* [5]. Population trends for all beaked whale species are classified as unknown on the IUCN Red List (with the exception of three species with signs of decreasing trends). One known issue is the sensitivity of this taxonomic group to certain types of noise generated by human activities [6, 7], suggesting the need for protective measures. However, the lack of data on most aspects of beaked whale life history and ecology—abundance, distribution, habitat preferences, mating and population dynamics—impedes the adoption of effective conservation and management strategies.

Beaked whales dive for up to an hour to depths of 500 m or more in search of deep-sea prey [8], recovering briefly at the surface between these deep dives [9]. This limits visual sightings and complicates traditional visual surveys, particularly as weather conditions deteriorate [10]. During foraging dives, beaked whales produce short, highly directional echolocation clicks to locate prey and navigate, with rapid and weaker pulse sequences, known as buzzes, used during prey capture efforts [11]. Clicks, produced at regular time intervals, have a characteristic frequency-modulated upsweep that distinguishes them from clicks of other toothed whale species [12]. These frequency-modulated clicks have species-specific differences in peak frequency, bandwidth, duration, spectral content, and inter-click interval [13], which allows monitoring of beaked whale species using passive acoustic monitoring (PAM) methods [14].

PAM has greatly aided the study of beaked whale populations, leading in one occasion to the evidence of a new beaked whale species [15] and addressing wide range of ecological questions, such as spatial and temporal distributions [14, 16], population differences [17], habitat use [18], and estimating animal densities [19], as well as the effects of anthropogenic activities and possible mitigations [20]. Advances in PAM techniques also enabled the collection of extraordinarily big datasets over longer periods of time, allowing year-round monitoring of remote regions to assess population trends [19]. However, efficient methods for automating the analysis of these large datasets are needed to reduce the effort in identifying and monitoring beaked whale species.

Most existing automated approaches for analyzing acoustic data involve two stages: a detection phase that finds sounds of interest from the recordings, and a classification phase in which detected sounds are assigned to specific categories (e.g., species) based on distinctive acoustic features. Automatic detectors have been developed to identify beaked whale acoustic events that meet certain heuristics [13, 21–23], but classification to species is often still manual and time-consuming [13, 24], or inaccurate with high false positive and missed detections if automated [25, 26]. Several machine learning techniques have emerged as effective tools for beaked whale species-level classification, by learning patterns from manually developed training sets [26–29] or groups of similar click types identified using unsupervised clustering algorithms [30–32]. By deriving clusters of similar click types based on the similarities of selected click features, unsupervised learning has automatically generated large training sets needed to teach supervised learning algorithms for species classification [29, 33]. This approach has helped discover and learn known and novel signals, and the acoustic identification of species previously not classifiable [31, 34, 35], allowing for realistic analysis with evolving knowledge of species acoustics recognition.

Machine learning algorithms designed to generalize and perform on a broad range of species may impair classification performance for particular species, which is detrimental to properly estimate animal densities or understand acoustic behavioral changes. Beaked whales dive in small groups, have highly directional clicks, and produce clicks at a slower rate than other species such as delphinids, therefore, detected acoustic events are often short in duration (i.e., ephemeral), and unsupervised clustering algorithms used to group similar signals may overlook them if they overlap in time with other species that dominate the recordings. When estimating local densities of beaked whales from acoustics, a detection probability for signals received above a particular amplitude threshold is necessary [19, 36]. The challenge with beaked whales is that the ephemeral nature of their acoustic encounters often leads to those occurrences being missed above the selected thresholds. This results in an undercounting of beaked whales and potential bias towards larger groups or deeper dives, when more or higher amplitude clicks are available. Similarly, behavioral studies assessing the effects of noise exposure may be bias towards these cases when classification is more reliable, as noise-exposed beaked whales have shown acoustic behavioral changes [6]. Animals interrupt foraging [e.g., 37–39], leading to shorter acoustic events that are more challenging to detect. Therefore, when using acoustics to accurately estimate beaked whale densities and the effects of noise exposure, detection and classification methods must identify clicks from ephemeral acoustic events, even in the presence of more acoustically dominant species. The generalization strength of machine learning techniques to classify a broad range of species may not be appropriate for these particular population studies, and a targeted species classification pipeline may help minimize bias by improving the detection of non-dominant signals, i.e. rarer signals.

The present study demonstrates that tailoring a deep neural network (DNN) to a subset of data focused on a specific taxonomic family, in this case beaked whales (family Ziphiidae), resulted in greater overall accuracy, particularly for ephemeral acoustic events. Building on current methods [31, 33–35], we developed a classification pipeline consisting of a combination of unsupervised clustering with a DNN trained narrowly on beaked whale signal types to improve species classification performance. A comprehensive library of the dominant click types for six beaked whale species in the western North Atlantic is provided: *Ziphius cavirostris*, *Mesoplodon densirostris*, *M*. *europaeus*, *M*. *bidens*, *M*. *mirus*, and the unknown signal type named “Beaked Whale Gulf” (BWG). Using this approach, we provide fine-scale detection and classification of beaked whale species, which can allow efforts to advance the study of populations in long-term monitored regions. The training data is publicly available in the HARP North Atlantic Beaked Whales repository hosted by Dryad. We aim to start the creation of a central database to improve rare species detection.

## Materials and methods

### Overall workflow

**Fig 1:**
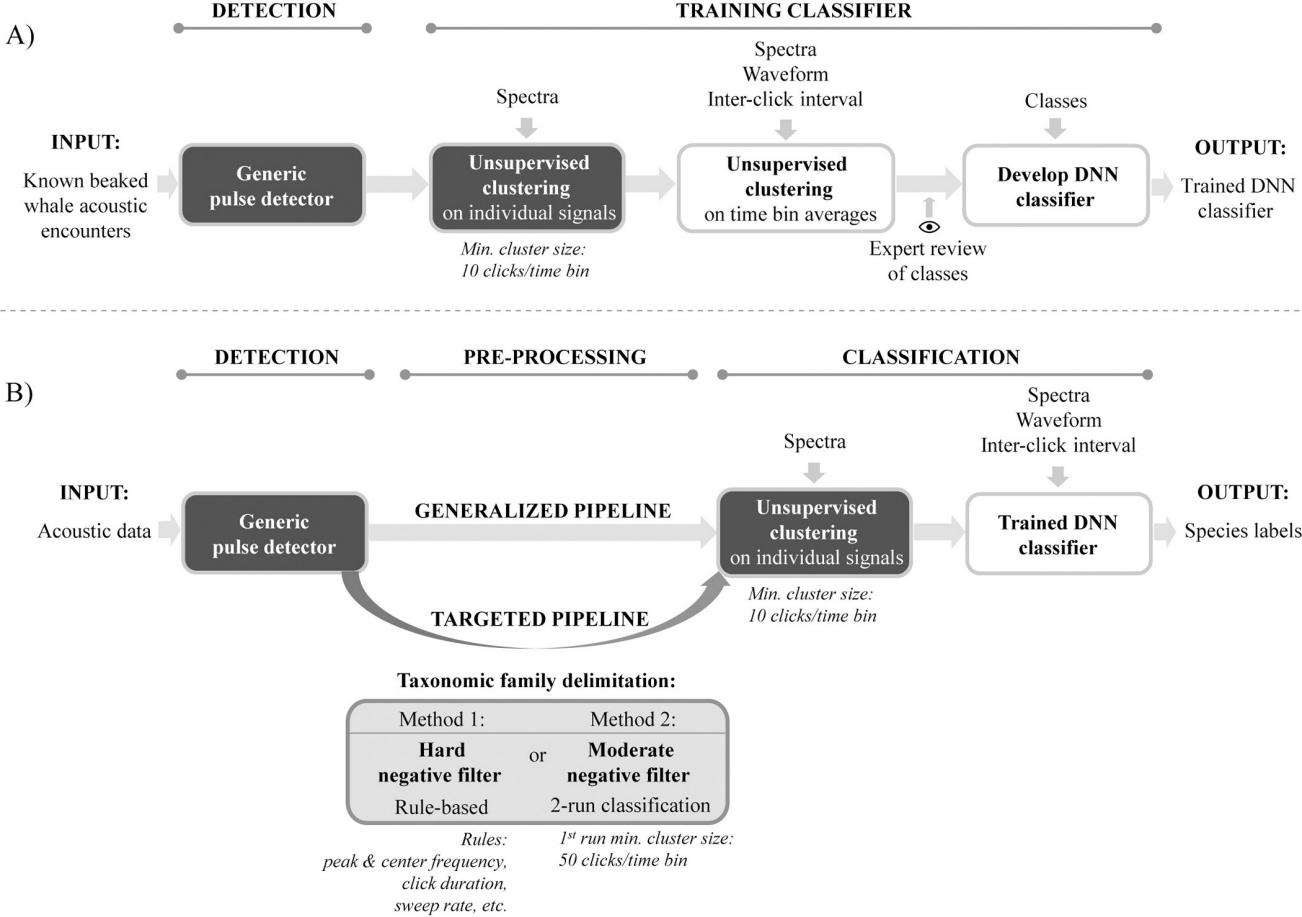
Overview of the workflow for a) training and b) applying a generalized and targeted species classification pipeline. Boxes indicate automated algorithms, in black are the standard steps between workflows and in gray is an additional step to delimitate data to a target group.

We provide an overview of the processes involved in machine learning for training and implementing a generalized and targeted species classification pipeline (**Fig 1**). All workflows included automated stages for signal detection, clustering and classification. The training workflow had three main steps: 1) a generic detector to detect pulses from audio data segments with known occurrences of beaked whales, 2) unsupervised clustering to identify distinct signals within a time window, followed by another pass of unsupervised clustering to group similar clusters into signal types, 3) human guidance for signal categorization to classes, and 4) development of a DNN with a training dataset (**Fig 1A**). The implementation of the trained DNN for a generalized classification of sounds included three main parts: 1) a generic pulse detector, 2) unsupervised clustering to identify distinct signals within a time window, and 3) a trained DNN to classify clusters within each time window, returning labels for each individual detection forming a cluster. For a targeted species DNN pipeline, a phase was added prior to clustering to automatically delimitate signals of interest (e.g., taxonomic family) and remove non-targeted signals (referred as negatives). Two delimitation methods were proposed a) a hard negative filter based on signal characteristics rules and b) a moderate negative filter that passed through two runs of unsupervised clustering and classification with a trained DNN, where the first run identified and excluded dominant non-beaked whale detections from later steps (**Fig 1B**). All steps were carried out with user-interfaces within the publicly available MATLAB-based (Mathworks, Natick, MA, USA) open-source software *Triton* [40].

### Passive acoustic monitoring data

Acoustic data were collected using bottom-moored High-frequency Acoustic Recording Packages (HARPs) consisting of a calibrated hydrophone suspended about 10–30 m above a packaged data logger, batteries, flotation, acoustic release, and ballast weight system [41]. Most recently deployed devices had a single omnidirectional sensor (ITC-1042), while older deployments used a bundle of low frequency (Teledyne Benthos AQ-1) and high-frequency (ITC-1042) sensors (**S1 Table**). The audio was digitized with 16-bit resolution at a 200 kHz sampling rate, and all device configurations provided an effective frequency bandwidth from 10 Hz to 100 kHz. Representative hydrophone systems were calibrated at the U.S. Navy Transducer Evaluation Center in San Diego, CA, to verify laboratory calibrations.

**Fig 2:**
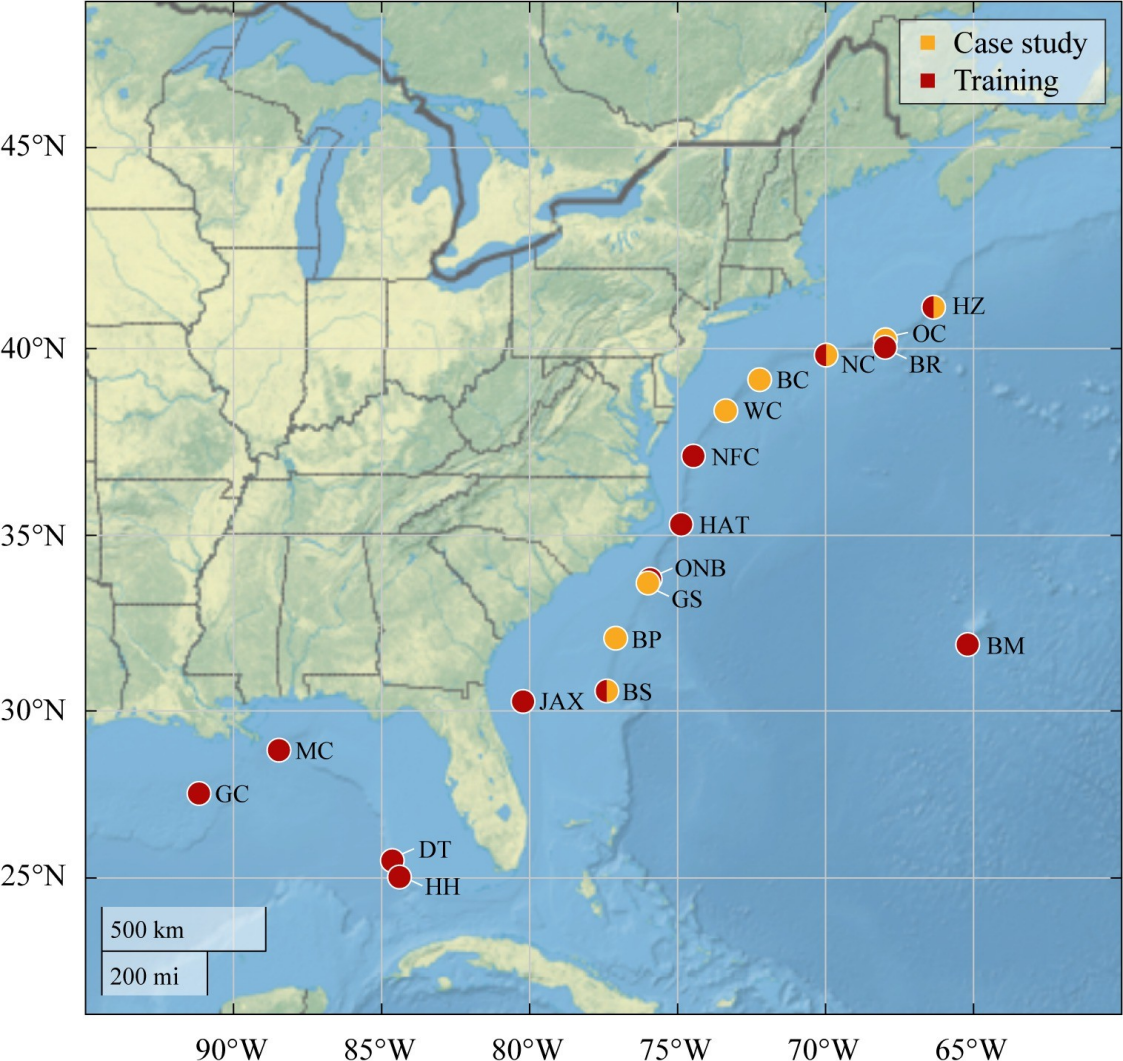
Map of recording locations. Map showing the latitude and longitude locations of all passive acoustic monitoring sites. Sites used in the representative training dataset are shown in red; sites included in the case study dataset are shown in yellow. Site abbreviations in the western North Atlantic: Heezen Canyon–HZ, Oceanographer Canyon–OC, Bear Seamount–BR, Nantucket Canyon–NC, Babylon Canyon–BC, Wilmington Canyon–WC, Norfolk Canyon–NFC, Hatteras–HAT, Onslow Bay–ONB, Gulf Stream–GS, Blake Plateau–BP, Bermuda–BM, Blake Spur–BS, Jacksonville–JAX. Site abbreviations in the Gulf of Mexico: Mississippi Canyon–MC, Green Canyon–GC, Dry Tortugas–DT, Howell Hook–HH. Map land cover, shaded relief, and ocean-bottom relief were obtained from Natural Earth (http://naturalearthdata.com/).

Acoustic data with known occurrences of beaked whales from nine sites in the western North Atlantic and four sites in the Gulf of Mexico at depths ranging from 450 to 2085 meters were used to train a DNN (**Fig 2 and S1 Table**). A case study was conducted using two months of data from seven sites in the western North Atlantic to evaluate the performance of a targeted species classification pipeline in detecting ephemeral acoustic events by comparing the labels produced by the classifier against traditional manual annotations. Additionally, at site Wilmington Canyon (WC) of the case study dataset containing a larger variety of beaked whale species, the performance of two targeted species pipelines using a hard negative filter and a moderate negative filter to delimit data were compared with the performance of a generalized species pipeline.

#### Dataset with known beaked whale occurrence

Acoustic events with known beaked whale occurrences documented on previous monitoring efforts were used to create a training dataset for a DNN classifier. Beaked whale occurrences were documented using a standard method for detection from PAM data [13]. The method included a Teager-Kaiser energy detector and a set of rules based on common signal characteristics of beaked whale species [13]. All signals had to meet the following criteria: a minimum duration of 355 μs, a peak frequency of at least 32 kHz, a center frequency of at least 25 kHz, a frequency-modulated upsweep with a sweep rate of at least 23 kHz/ms, and a waveform envelope that sloped positively during the first 0.1 ms and stayed at or above 50% energy for at least the proceeding 0.1 ms. Clicks were then analyzed at 75-second intervals and kept for classification if intervals had at least seven candidate signals or if 13% of the signals met all criteria. Finally, acoustic events were manually classified to species based on the inter-click interval (ICI) and the overall shape of the mean spectra of all clicks within an encounter.

#### Manually classified case study dataset

For the case study dataset a generic pulse detector with the same settings described below was implemented and non-typical beaked whale signals were removed using the common set of rules described above. All detections were manually classified to beaked whale species using *DetEdit* [42], a MATLAB-based editing open-source software. This provided interactive displays to compare detections, with information about, for example, individual or averaged waveforms and spectra of selected detections, ICI and long-term spectrograms to place detections in a broader context.

### Generic pulse detector

A generic pulse detector was the first automated algorithm in all workflows, implemented using the publicly available *SPICE-Detector* remora package [33] in *Triton*. Applying a band-pass filter to all recorded data, potential toothed whale clicks were detected if the acoustic pulse energy was over a received sound pressure level (SPL) threshold and if the peak frequency and durations of the signals were within typical ranges for most species in this group. The detector’s specifications included a fifth-order Butterworth filter with a high passband between 5 and 100 kHz to remove low-frequency noise. Signals were required to have a minimum received SPL of 118 dB peak-to-peak re 1μPa^2^ to maintain a constant detection range around the recorder, and were kept if the durations were between 30 and 1200 μs and the peak frequencies were between 5–100 kHz, which is within the ranges for the majority of clicks. If there were less than 100 μs between signals, they were merged. Each signal start and end time was defined by finding the first and last sample on each side of the main peak where energy was greater than the 70^th^ percentile for the entire high-energy event. Each signal time series was Hann-weighted and zero-padded to 400 points before using a 400-point discrete Fourier transform to compute spectra, resulting in a standard interpreted bandwidth of 50 Hz/frequency bin.

### Unsupervised clustering: Identification of distinct signal classes

An unsupervised clustering method designed by Frasier *et al*. [31] and available from the *Cluster-Tool* remora package [33] in *Triton* separated detections into distinct signal types. Signal types were identified in successive 5-min time windows, providing intervals both short enough to limit the number of classes detected concurrently and long enough to allow the clustering algorithm to identify meaningful groups. Signals within each time bin were compared for spectral shape similarities using pairwise distance metrics [43] and clustered into similar types using the agglomerative Chinese Whispers algorithm [44]. Spectra were truncated at 10 and 90 kHz and normalized to [0, 1] amplitude range for similarity metric calculation, creating a [0, 1] edge weights matrix. For each 5-min bin, a network was built at the start of the clustering phase, with detections as nodes and edge weights connecting each node. To identify distinct clusters, weak edges below a certain threshold were removed to filter out “noisy” edges. Although edge pruning is a standard strategy for revealing the underlying structure, it may remove highly distinct signals from any final clusters. Taking into account cluster consistency and avoiding unnecessary splitting of strongly connected clusters, an edge pruning threshold of 80% was selected after evaluating several clustering iterations with varying threshold from 70% to 95%. The agglomerative clustering algorithm divided clusters of similar nodes within a maximum of 25 iterations and a maximum network size of 40,000 clicks for each 5-min bin. The final partition for each bin was chosen based on the highest average normalized mutual information score [45], which compares clusters across multiple partitions to decide on consistency of types. Small clusters, likely capturing ephemeral beaked whale events, were allowed to form within a bin with a minimum of 10 clicks per cluster, providing enough data for a unique signal type to be identified.

Frasier *et al*. [33] successfully classified odontocete species based on three signal features describing click duration, spectral shape, and clicking rate. The same features were computed for each cluster formed at each 5-min bin and used for classification in the following phase. Averaged features included a mean spectrum of all clicks within a cluster, mean waveform envelope of clicks, and the ICI distributions from sequential clicks. To calculate mean spectra, power spectral density of clicks was computed, converted to a dB scale, and normalized to the [0, 1] range. Normalized spectra were then averaged to obtain the mean normalized spectra on a dB scale. To address missed detections and gaps between clicking, ICI distributions were limited to 0.8 seconds within typical ranges for most toothed whale species, and a modal distribution was computed to characterize a consistent clicking rate.

### Development of training dataset for ephemeral signals

Bulk unsupervised clustering of all detections does not guarantee quality training examples. Therefore, this study aimed to create a high-quality beaked whale training set for a DNN classifier. This involved detecting signals from known beaked whale acoustic events, unsupervised clustering of high-quality events, training a DNN, and evaluating its performance on an independent test set. The process ensured quality training examples and a representative dataset for the DNN.

#### Building a representative dataset of signal classes

The generic pulse detector detected signals during time intervals with known occurrences of beaked whales. These acoustic events included both single-species and mixed-species beaked whale events, detected alongside other delphinids or noise sources, requiring the separation of signal types. A two-step unsupervised clustering approach was used to identify dominant signal types representative of the major signal classes observed within each acoustic event (further details in Frasier *et al*. [31]). First, using the same settings as described before in the unsupervised clustering section, signals were clustered within 5-min bins, with a higher edge pruning set at 95% to generate more consistent clusters (following the approach of Frasier *et al*. [31]). Mean spectra, waveform envelopes, and the mode of the ICI distributions were calculated for each 5-min bin cluster formed and used as summary cluster features for the second phase. In the second round, the same algorithm was used to the bin-level clusters to identify dominant signal types across all bins in each instrument deployment independently. Similarities were computed by comparing the mean spectral shape and the mean waveform envelope using pairwise distances. Euclidean distances between modal ICIs were calculated to determine ICI distance values and converted into a similarity metric. The similarity scores were joined and used in the Chinese Whispers clustering algorithm to allocate clusters with the same settings as in the first clustering step, though retained clusters were required to have at least 5 bin-level clusters. To improve the number of clusters formed of the BWG signal type, the joined similarity metric was computed only by comparing the mean spectral shape and the modal ICI distributions, excluding the waveform envelope due to high variability in click duration. The collection of dominant signal types was visually examined by analysts (ASB, AID, JST, and LMB) and attributed to known species or sound source classes. Multiple clusters from a deployment were allowed to contribute to a signal class on the premise that click types show natural variability when recorded, and the clustering parameters were set to err on the side of formation of more small clusters, which may represent different versions of the same category. The alternative, formation of fewer clusters, can lead to incomplete separation of similar types.

#### Training a deep neural network

A DNN was trained with the representative signal classes using the publicly available *Triton* remora *Neural Net Tool* package [33]. The dataset was partitioned into training, validation, and test sets, with the training set for training the network, the validation set for assessing the network’s fit and fine-tune parameters across training iterations, and the test set for evaluating its performance on novel data. For this data partition phase, individual detections from each class were sorted into encounters–times when detections were not separated by more than 15 minutes–and randomly assigned to the train/validate/test sets in the proportions 70/10/20, without partitioning detections from the same encounter within sets. Several classes had far more examples than others (**Table 1**) and a balanced collection of 1,500 examples was obtained per class to improve the network’s performance, as classification of rare species is crucial in this framework. Examples from well-represented classes were subsampled and examples from minority classes were augmented by adding Gaussian noise to obtain the same number of examples. The balanced collection was randomly subdivided into 1,000 training examples and 500 testing examples, with the training examples further partitioned at random into training and validation sets in 80/20 proportions. The summary features of each cluster example were normalized on the [0, 1] range. The DNN architecture was created using the Deep Learning Toolbox in MATLAB, consisting of an input layer, four 512-node fully-connected layers with leaky rectified linear unit (ReLU) activations [46] and 50% dropout between layers, as well as a softmax output layer that generated a vector of probability scores over class labels [47]. Deep learning was completed with a batch size of 100 examples per iteration, a constant learning rate of 0.0003 and a maximum of 15 training epochs. To prevent overfitting, learning was stopped early if there was no performance improvement on the validation set between iterations after three training epochs of patience.

**Table 1:** List of labels assigned to classes representing signals from North Atlantic beaked whale species and other signal sources. Class summary with abbreviation ID, description of the attributed class–species, taxonomic family, genus, or common descriptive features–and total number of bin-level clusters per class, median and interquartile range of detections per cluster, and sites where clusters were acquired. Site abbreviations: in the western North Atlantic, Heezen Canyon–HZ, Bear Seamount–BR, Nantucket Canyon–NC, Norfolk Canyon–NFC, Hatteras–HAT, Onslow Bay–ONB, Bermuda–BM, Blake Spur–BS, Jacksonville–JAX; in the Gulf of Mexico: Mississippi Canyon–MC, Green Canyon–GC, Dry Tortugas–DT, Howell Hook–HH.

Class ID	Description	Total # clusters	Signals per cluster median (IQR)	Total # clusters per monitoring sites												
				HZ	BR	NC	NFC	HAT	ONB	BM	BS	JAX	MC	GC	DT	HH
BWG	Unknown species named as Beaked Whale Gulf	29	10 (9)								7			6	16	
Mb	*Mesoplodon bidens*	1,852	50 (116)	1,121		23	704					4				
Md	*Mesoplodon densirostris*	1,035	38 (90)				6	5	2	399	558	3		50	3	
Me	*Mesoplodon europaeus*	8,687	51 (125)					120	3,073	2	765	28	605	539	2,559	996
Mm	*Mesoplodon mirus*	1,115	43 (116)	49	227	152	687									
Zc	*Ziphius cavirostris*	10,917	58 (139)	649	232	3	597	2758	20	25	187		84	179	4,447	1,738
De spp	Unspecified species from the family Delphinidae	25,316	95 (438)	3,295	490	312	2,793	15,367	140	132	145	121	769	179	1,062	
Gg	*Grampus griseus*	2,039	69 (218)	146	45				66						79	1,703
Ko spp	Unspecified species from the genus *Kogia*	50	25 (22)						19		18					13
Pm & Boat	Detections with freq. < 20 kHz, vessels and *Physeter macrocephalus*	10,411	46 (108)	2,262	547	91	880	1,809	1,196	248	76		1,170	646	900	586
ES ping	Miscellaneous echosounder pings	262	41 (43)		170			92								

#### Performance evaluation

The DNN’s performance was evaluated throughout training and testing by visualizing confusion matrices and computing two metrics less sensitive to class imbalance [48]: precision (true positives / (true positives + false positives)) and recall (or sensitivity: true positives / (true positives + false negatives)). Precision indicates the likelihood that the prediction was correct, while recall assesses the likelihood that the model will correctly predict or retrieve that label.

### Targeted species classification pipeline

After training the DNN, the classification pipeline was used to automatically detect and classify data from a case study dataset. A targeted species classification pipeline was developed and compared to a generalized species pipeline. Both workflows applied a generic pulse detector, unsupervised clustering and the trained DNN for cluster labeling (**Fig 1B**).

### Taxonomic family delimitation

The targeted pipeline differed from the generalized pipeline in that it included a step for taxonomic family delimitation before clustering to exclude non-beaked whale detections (i.e. negatives) dominating the recordings. This allowed for more exact grouping of signals of smaller and varying sizes. Two delimitation methods were tested (**Fig 1B**) and available from the *Taxonomic-Family-Delimitation* remora package in *Triton*: a) a hard negative filter based on commonly used rules to identifying beaked whale signals from passive acoustic data [e.g., 13, 14, 16], and b) a moderate negative filter that underwent two iterations of unsupervised clustering and classification with a trained DNN (**Fig 1B**). The hard negative filter discriminated beaked whale clicks using the same set of rules as the manually-classified species datasets. The moderate negative filter was less restrictive and consisted of two iterations of unsupervised clustering and classification with the trained DNN, with the first iteration removing the most dominant non-beaked whale detections. In the first iteration, unsupervised clustering allowed only large-sized clusters to form within a 5-min bin with a minimum of 50 clicks per cluster and a pruning threshold of 95%, providing enough data to likely capture the dominating species events while being computationally efficient to cluster the large amount of detections. Next, the trained DNN categorized clusters, and those with at least 50 clicks and a label other than beaked whale were removed. This initial pass automatically eliminated a large number of delphinid signals and noise sources. In the second iteration, unsupervised clustering was implemented using the same settings as in the generalized pipeline or the targeted species pipeline with the hard negative filter, allowing small clusters to form within a 5-min bin with a minimum of 10 clicks per cluster, and finally, the trained DNN classified clusters.

#### Performance evaluation

The efficacy of the targeted pipeline in detecting ephemeral events and rare beaked whale species was assessed using the case study data. Manual species labels were used as true classes and compared to the trained DNN classifications in 5-min bins. The targeted pipeline with the hard negative filter was compared with the manual classification across all sites of the case study dataset to ensure classification comparability of ephemeral events, as both methods used the same rules to remove non-typical beaked whale signals. To quantify misclassifications, the class category “No label” was assigned to bins in which no labels were applied during manual labeling or where no clusters formed and no labels were given. The number of detected clicks within 5-min bins and the classification prediction probability score were used to assess performance per species and site. Performance was also assessed by visualizing confusion matrices, computing precision and recall rates, and plotting acoustic features of the misclassified detections. Additionally, at site WC of the case study dataset, which contained a larger variety of beaked whale species, the performance of both delimitation methods in the targeted pipeline were compared with the generalized pipeline. Confusion matrices, precision and recall rates, and acoustic features of the misclassifications were also used to evaluate performance on a species-by-species basis.

## Results

### Training dataset of representative signal classes

**Fig 3:**
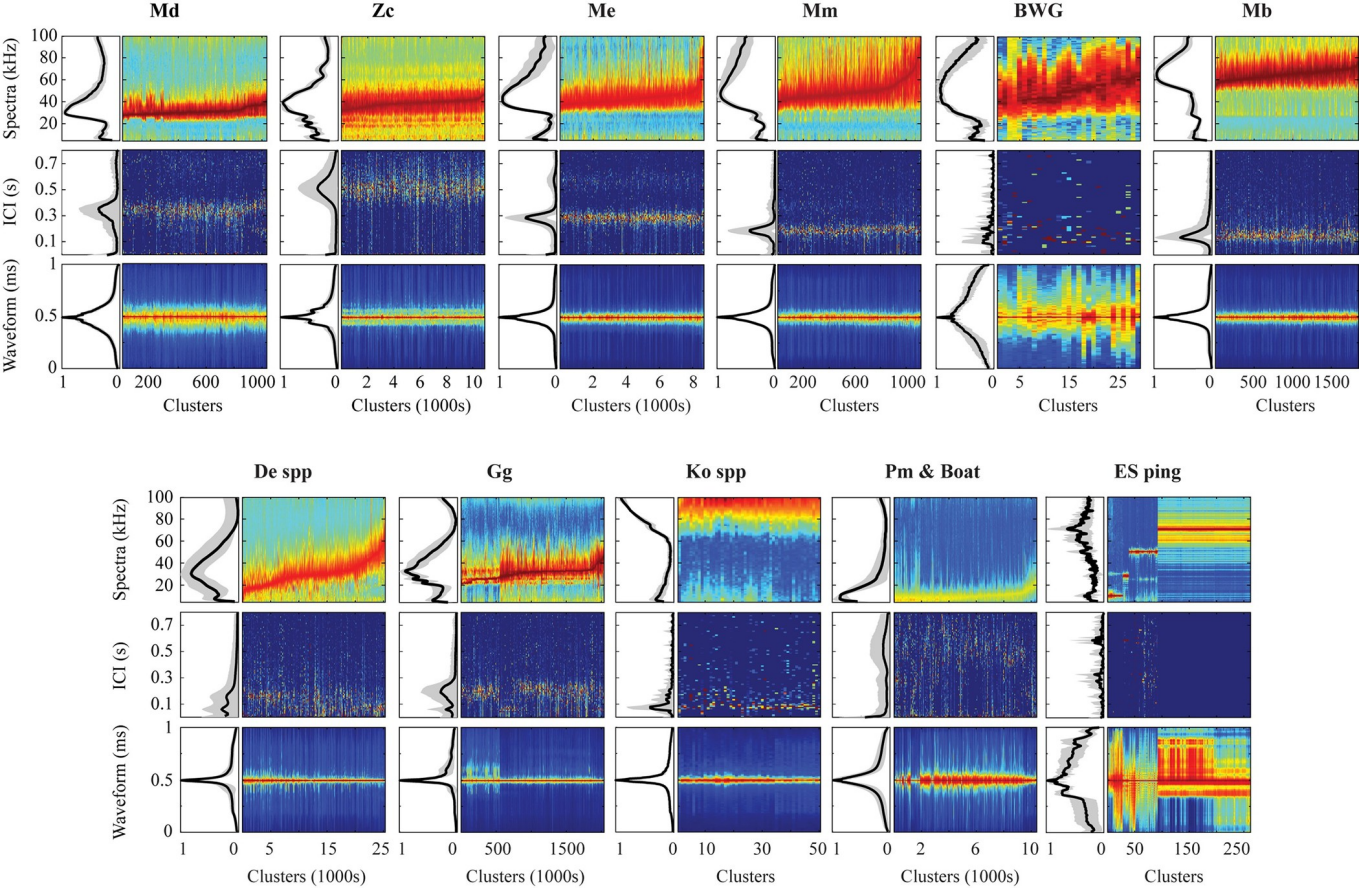
Signal classes formed using unsupervised clustering at a 5-minute bin level based on spectra, inter-click interval (ICI), and waveform envelope. For each signal class, the top panels depict the mean and standard deviation of spectra among all clusters (top left) and concatenated mean cluster spectra (top right); the middle panels depict the mean and standard deviation of ICI distributions among all clusters (middle left) and concatenated cluster ICI distributions (middle right); and the bottom panels depict the mean and standard deviation of waveform envelops among all clusters (bottom left) and concatenated mean cluster waveform envelope (bottom right). Concatenated clusters have been sorted by peak frequency. Color map is normalized amplitudes on a scale from 0 (dark blue) to 1 (dark red). Refer to **Table 1** for abbreviation IDs.

A total of 11 classes of clustered signals at a 5-min bin level were generated (**Table 1 and Fig 3**), with six classes representing distinct beaked whale species. *Mesoplodon densirostris* (Md) signal type was derived from clicks collected at two sites in the Gulf of Mexico, four sites in the middle and southern parts of the western North Atlantic, and from the Bermuda region in the central North Atlantic (**Table 1**). All clicks had highly similar spectral shapes, but the frequency onset and peak frequency varied by region (**Fig 3 and S1 Fig**). Amongst the sites with the most *Mesoplodon densirostris* detections the frequency onset of clicks collected in the Bermuda region was higher (~ 25 kHz) than those recorded in the western North Atlantic (Blake Spur) and the Gulf of Mexico (about 21–22 kHz). The peak frequency of clicks collected in the Bermuda region varied between 29.5 and 36.5 kHz, whereas the peak frequency at the BS and GC sites was between 31–32.5 kHz (**S1 Fig**). Due to their much shorter duration (less than 250 μs), clicks collected at GC site were different from those collected in the other two regions. As a result, this class represented certain intraspecific variability. For the other beaked whale species, no regional differences in the spectra, waveform and ICI were found. Clusters of the *Ziphius cavirostris* (Zc) click type were formed by the unsupervised clustering process from all sites except JAX, characterized by a peak frequency at around 40 kHz, three distinct secondary peaks (at 19, 24, and 70 kHz), and an averaged modal ICI of 0.51 s (**Table 1 and Fig 3**). *Mesoplodon europaeus* (Me) and *M*. *mirus* (Mm) click types differed consistently in the average modal ICI. Mm had a shorter modal ICI than Me, which was about 0.19 s and 0.28 s, respectively. The frequency onset of both click types was around 30 kHz; but the Me click type reached its highest amplitude at 40–44.5 kHz at a steeper rate than Mm click type, which reached its peak amplitude near 47 kHz. Moreover, *Mesoplodon europaeus* clicks often decreased in amplitude at a steep rate after 45.5 kHz (**Fig 3**). Both click types presented a characteristic secondary frequency peak (Me at 23.5 kHz and Mm at 24.5 kHz). Clusters of the *Mesoplodon mirus* click type were formed by the unsupervised clustering only in the sites north of Hatteras (HAT) in the western North Atlantic (**Fig 1**), whereas *Mesoplodon europaeus* clicks were formed from all sites except the northern sites (**Table 1**). The unknown species producing the BWG signal was exceptionally rare and challenging to cluster when 5-min bin clusters were built using the averaged spectral characteristics. Knowing that the click duration of this signal type is particularly long and distinct from the other signals, a separate approach was applied to collect clusters representing this class from the known acoustic events, which were built simply using the average waveform envelope rather than the averaged spectra. A few clusters collected from two sites in the Gulf of Mexico region and the BS site in the western North Atlantic’s southern region were used to create the BWG click type (**Table 1**). All clicks had a particularly long duration exceeding 0.5 ms, with an averaged modal ICI of roughly 0.1 s. The spectral shape displayed a gradual increase in amplitude from the frequency onset at around 22–24 kHz to the peak amplitude that varied between 40–60 kHz (**Fig 3**), and had a secondary peak at 17 kHz. *Mesoplodon bidens* (Mb) click type was formed by the unsupervised clustering from four sites across the western North Atlantic, and it had the most distinctive spectral shape of any beaked whale type, with energy distributed across a wide band from 50 kHz to 90 kHz, and an average modal ICI of 0.13 s (**Table 1 and Fig 3**). This study did not include a signal type for *Hyperoodon ampullatus*, which is commonly found in the polar and subpolar regions of the North Atlantic, primarily in Canadian waters in the North Atlantic [49]. Only two sightings were reported in the early 1980s off New Jersey and the Grand Banks, corresponding to the northern sites of this study, and a recent survey did not detect them in this area [49]. PAM recordings for this species exist in more northern locations, but due to different sampling rate, and that the species has a lower peak frequency than the other beaked whale species in the North Atlantic [16], the hard negative filter of the targeted pipeline was not optimal for detecting them and there were no acoustic encounters available to build a signal type class for the training dataset.

Non-beaked whale signals were identified during unsupervised clustering and assigned to classes of other odontocete species and noise sources to reduce the possibility of them being misclassified as beaked whales. Non-beaked whale odontocete species were divided into three click type classes: a broad class encompassing multiple species of the family Delphinidae, a class for narrow band high frequency clicks of the genus *Kogia*, and a class for *Grampus griseus* (Gg) clicks. Early training experiments revealed that providing one unique class for all delphinid species caused a considerable number of *Grampus griseus* clicks to be misclassified as beaked whales; as a result, Gg clicks were defined into one unique class. Diverse echosounder pings (operating at 9.5, 28, 50.5, and 71 kHz) were categorized into a single class, as were low-frequency signals below 20 kHz associated with ship cavitation noise and *Physeter macrocephalus* (Pm) clicks.

The number of bin-level clusters varied between classes, ranging from large numbers for more common signals such as *Ziphius cavirostris*, *Mesoplodon europaeus*, and Delphinidae spp. to a small number for the BWG class and moderate numbers for *Mesoplodon mirus*, *M*. *densirostris*, and *M*. *bidens* (**Table 1**). To increase signal class variability, the majority of classes were formed of bin-level clusters from more than three sites, except for the echosounder class, which consisted of clusters from two sites. The dataset collected is accessible under the HARP North Atlantic Beaked Whales repository hosted by Dryad to facilitate the representation of rare species. The collection provides a snippet of acoustic data centered on each detection and organized by signal class.

### DNN performance during training and testing

**Fig 4:**
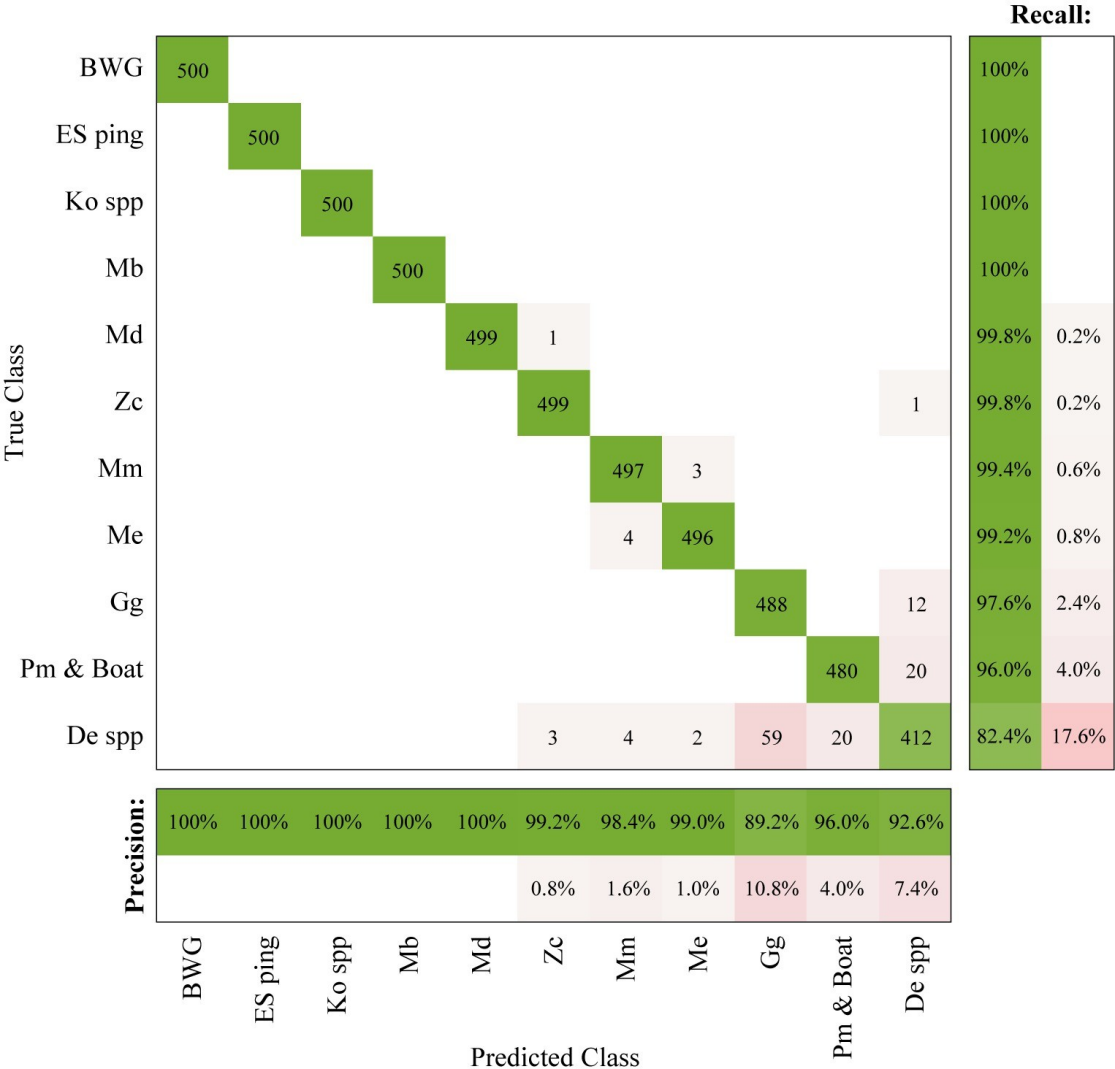
Confusion matrix for the DNN classifier on the balanced test set. Each class consists of 500 cluster examples formed at a 5-minute bin level. Values in the matrix show the total number of examples classified, on the right bar the recall rate, and on the bottom bar the precision rate. Refer to **Table 1** for abbreviation IDs.

The training of a DNN using the balanced training set (N = 1,000 bin-level clusters per class) required 10 learning epochs. The network classified the balanced test set of 500 cluster examples per signal class with an overall accuracy of 97.7%. As expected, confusion was higher for non-beaked whale classes since multiple species of delphinids were grouped into one class to prevent spurious assignments of unrepresented non-target signals to beaked whale categories (**Fig 4**). Confusion was minimal for all beaked whale species, with the BWG and *Mesoplodon densirostris* click type in particular being correctly classified with precision and recall of 100% in all cases. Several clusters for *Ziphius cavirostris*, *Mesoplodon bidens*, *M*. *europaeus*, and *M*. *mirus* click types appeared to be misclassified, but some of these clusters were correctly classified by the DNN and then incorrectly grouped during the unsupervised clustering step (**S2 Fig**). Yet, the performance for the beaked whale species was still as desired, with the lowest recall rate (>99%) exceeding the network’s precision (>98.4%). The Delphinidae spp. class had the lowest performance among the non-beaked whale classes, with a recall rate of 82.4% and precision of 92.6%, and the *Grampus griseus* click type had the lowest precision (89.2%). Due to the inclusion of multiple species in the Delphinidae spp. class, a non-cohesive class characterization may have resulted from resampling the data to achieve a balanced dataset.

### Case study: Classification performance of a targeted species pipeline for ephemeral beaked whale events

Five beaked whale species were manually identified with varying occurrences across the case study sites. These included *Ziphius cavirostris*, *Mesoplodon densirostris*, *M*. *europaeus*, *M*. *mirus*, and *M*. *bidens* (**S2 Table**). The BWG signal was not manually identified at any of the locations, nor was the set of rules of the hard negative filter optimal for detecting *Hyperoodon ampullatus* clicks [16]. Labels applied to each cluster formed at the 5-min bin level by the targeted species classification pipeline with a hard negative filter was compared to the manually identified species presence across sites (**Table 2**) and per site (**S3 Table**). The DNN applied labels to clusters of clicks identified within 5-min time bins, while manual labeling was conducted at the detection level, taking into account the context of clicks around an acoustic event across multiple bins. When only one species was manually labeled per bin, but the classifier assigned multiple clusters of clicks with distinct labels, the classification performance was evaluated as multiple labels within that bin and counted as a bin for each related class in the confusion matrix. Similarly, if the classifier identified only one cluster and assigned one label but multiple species were manually recognized in one bin, each label was counted as a bin for each true class in the confusion matrix. The total number of false negative and false positive bins, as well as precision and recall rates were provided. Additionally, the performance metrics were provided without considering the multiple labels as a bin described previously. Instead, if the correct label was given to at least one detection with a bin, the bin was considered as correctly labeled; therefore, bins that were partially misclassified were not considered misclassifications, and the metrics were calculated considering only bins where none of the detections were assigned the correct class (referred as full-misclassified bins).

**Table 2:** Confusion matrix for the targeted species classification pipeline with a hard negative filter on the case study dataset. Values show the total number of 5-minute bins classified per class. Refer to **Table 1** for abbreviation IDs. Manually labeled bins are the true class. Bins with no clear assignment are indicated with the class abbreviation in italic. The total number of false negative (FN) and false positives (FP), precision and recall rates are shown per species and in parentheses when only full-misclassified bins (full bin) are considered.

		Predicted Class												# FN (full bin)	% Recall (full bin)
		Mb	Zc	Mm	Me	Md	BWG	De spp	Gg	Ko spp	Pm-boat	ES ping	*No label*		
**True Class**	Mb	**766**	1	14	1	2	6	27	1	3	2	-	30	87 (81)	89.8 (90.4)
	Zc	1	**1188**	20	13	6	20	61	20	1	1	3	12	158 (146)	88.3 (89.1)
	Mm	4	6	**315**	11	-	5	9	1	3	-	-	5	44 (31)	87.7 (91.0)
	Me	3	51	486	**2563**	4	82	32	3	7	-	1	-	669 (628)	79.3 (80.3)
	Md	-	-	-	1	**2**	1	3	-	-	-	-	-	5 (4)	28.6 (33.3)
	*Mm | Me*	-	1	32	5	-	3	2	-	-	-	-	-	43	
	*Md | Me*	-	-	-	1	-	-	-	-	-	-	-	-	1	
	*likely Me*	-	-	-	-	-	1	-	-	-	-	-	-	1	
	*likely Md*	-	-	-	-	-	1	1	-	1	-	-	-	3	
	*No label*	28	604	809	231	713	2671	4309	2195	165	22	97	4772	16616	
**# FP (full bin)**		36 (31)	663 (646)	1361 (1318)	263 (261)	725 (704)	2790	4444	2220	180	25	201	4819		
**% Precision (full bin)**		95.5 (96.1)	64.2 (64.8)	18.8 (19.3)	90.7 (90.8)	0.3 (0.3)									

The targeted species classification pipeline with a hard negative filter achieved an average recall rate of 75% of bins with beaked whale presence, with varying performance among species and sites. A proportion of the false negative bins were misclassified into other classes, and a lesser amount were unlabeled because these bins were not directly available to the trained DNN during the labeling step as a result of the clustering settings of a minimum number of clicks per cluster. *Mesoplodon densirostris* had the lowest recall rate at 29% (or 33% when considering only full-misclassified bins), but the other beaked whale species (*Ziphius cavirostris*, *Mesoplodon mirus*, and *M*. *bidens*) had recall rates above 87% (or 89% when considering only full-misclassified bins). *Mesoplodon europaeus* had the highest number of false negatives (with a recall of 79% and 80% when considering only full misclassified bins), with more than half of the misclassified bins assigned to *Mesoplodon mirus*. All misclassifications of this species as Mm occurred at the most southern locations, corresponding to the GS site and the BP site (**S3 Table**), where the abundance of *Mesoplodon europaeus* is known to be higher than at the central and northern sites.

**Fig 5:**
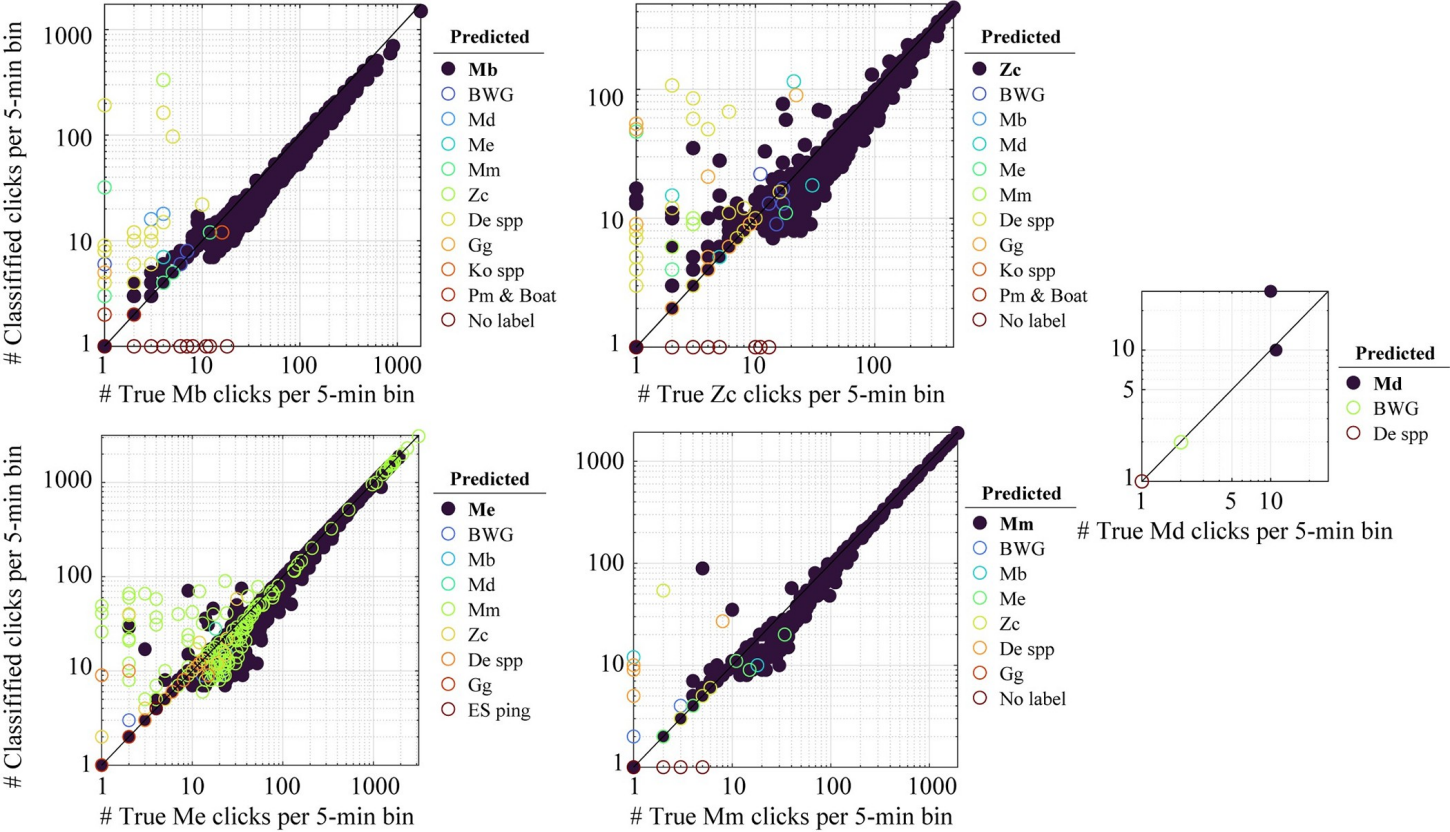
Correlation between the total number of clicks per 5-minute bins manually labeled to a class (true class) and total number of clicks classified to predicted class by targeted species pipeline with a hard negative filter in the case study dataset. Each point is a bin, and each subplot displays all bins manually assigned to a species, along with the class predicted by the DNN in color scale, with dark blue points representing true positives and other colored circles representing misclassified bins. The class abbreviation displayed in bold shows the true class of each subplot. Points along the diagonal show bins for which the number of manually labeled clicks were correctly predicted to a class. Above the diagonal, more clicks were predicted to a class than the true number of clicks per bin, whereas below the diagonal, less clicks were predicted than the total number of true clicks per bin.

*Mesoplodon densirostris* was the rarest of the five beaked whale species detected, and it was only manually identified in six 5-min bins at site BP (**Table 2**). The trained DNN correctly classified two bins from the manually identified events (with at least 10 clicks) as belonging to *Mesoplodon densirostris*, with one bin partially misclassified (three clicks) as Me. The remaining four bins (with fewer than two clicks) were mislabeled as BWG and Delphinidae spp. (**Fig 5**). All bins were correctly recognized to the species class for the other four beaked whale species when there were more than 20 clicks in each bin, except for multiple bins manually identified as *Mesoplodon europaeus* with varying numbers of clicks which were misclassified as Mm (**Table 2 and Fig 5**). In contrast, the trained DNN had a low misclassification of *Mesoplodon mirus* bins as Me. Overall, the majority of bins entirely misclassified occurred when there were fewer than 10 clicks associated with a beaked whale species as a result of prior criteria of cluster formation with presumably low-quality features for classification (**Fig 5**). When there were fewer than 10 clicks within a 5-min bin, no unsupervised clustering was applied and all clicks were averaged to extract the classification features. The trained DNN applied a label with respect to the averaged features from the clicks present in these bins, and if there was a mix of species, the averaged features probably diverged from the common learned features of the beaked whale classes and so misclassification occurred. Unsupervised clustering was applied if bins had more than 10 clicks, but clusters with fewer than 10 clicks were discarded from classification and so no labels were kept for the clicks within those clusters, therefore those bins remained unlabeled. Even when several clicks within a bin were correctly classified to the species class, there was a trend of missed or misclassified clicks within bins for all beaked whale species, where bins with fewer clicks had more clicks incorrectly labeled or unlabeled by the DNN, and the proportion of clicks correctly predicted increased when bins had more than 40–50 clicks (**Fig 5**).

**Fig 6:**
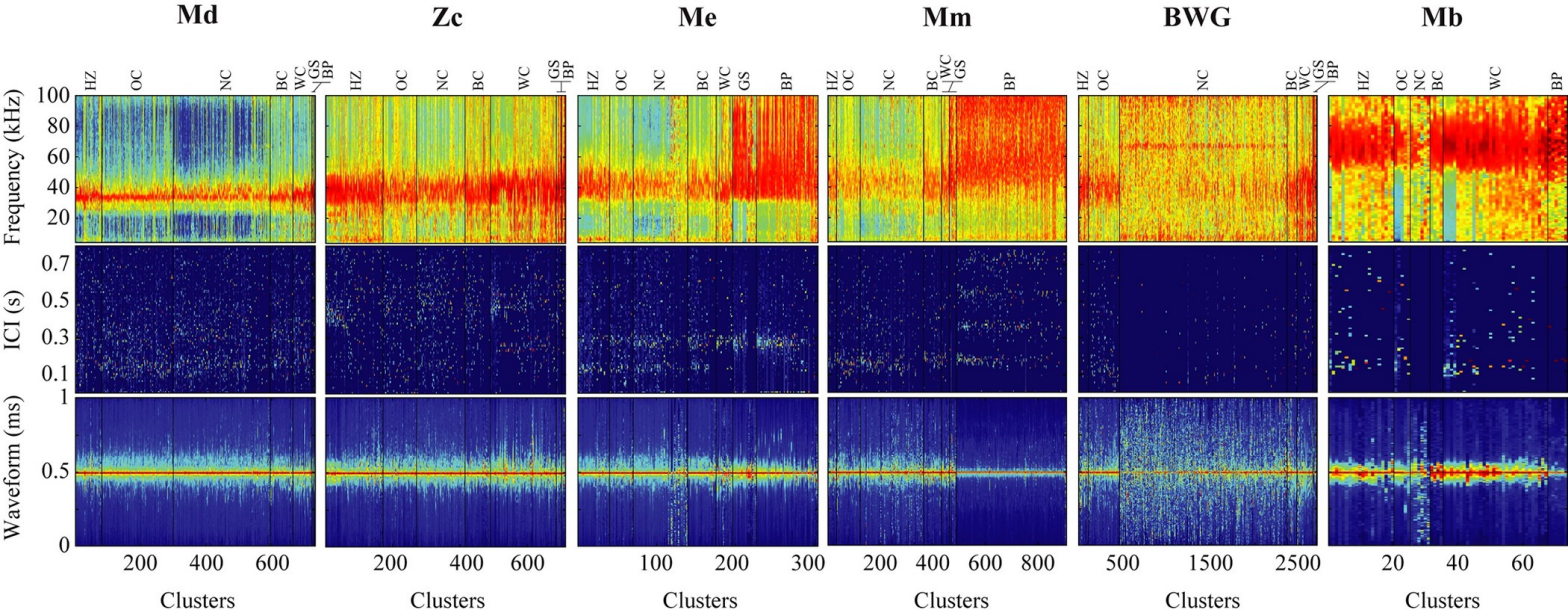
Case study dataset bins without a manual label classified by the targeted species classification pipeline with a hard negative filter. For each predicted beaked whale class, concatenated mean cluster spectra (top panel), concatenated cluster ICI distributions (middle panel), and concatenated mean cluster waveform envelope (bottom panel) are shown. Concatenated clusters have been sorted by site and classification prediction probability score. Color map is normalized amplitudes on a scale from 0 (dark blue) to 1 (dark red). Many of the clusters counted as misclassifications appear to have been correctly classified by the network, but were not labeled in the manual dataset. Refer to **Table 1** for abbreviation IDs.

The targeted pipeline with the hard negative filter had an overall lower precision than recall for the beaked whale species, with an averaged precision rate across all beaked whale species of 54%, due in an abundance of possible false positive bins for certain species and sites (**Table 2**). Rates varied from high precision above 90% for *Mesoplodon bidens* and *M*. *europaeus*, to moderate above 60% for *Ziphius cavirostris* and low below 20% for *Mesoplodon mirus* and *M*. *densirostris*. Rates were similar when considering only full-misclassified bins. The majority of false positive bins in this assessment were not manually identified with beaked whale presence, except for approximately 40% (n = 486) of the false positive bins classified as *Mesoplodon mirus* which were true *M*. *europaeus* (**Table 2**). Because the manual dataset only had labels for beaked whale species, the majority of unlabeled bins were most likely associated with other cetaceans and noise sources. However, the precision rates may be an underestimate as some evidence suggests that some true events may have been missed by manual review. The cluster averaged features of any bins categorized by the DNN as beaked whales and without a manual label were inspected (**Fig 6**). The lowest numbers of false positive bins with no manual label categorized by the trained DNN as beaked whales were at the two most southern sites, GS and BP (**Fig 6 and S3 Table**), and the highest numbers were at the northern site NC (**Fig 6 and S3 Table**). Cluster features resembled detections similar to those of the *Grampus griseus* click type, but without the characteristic lower peaks at 23.5 and 27 kHz. The majority of *Mesoplodon densirostris* false positive clusters corresponded to this type of signal, although they appear to have a frequency onset at around 21–22 kHz like *Mesoplodon densirostris*, the peak frequency is similar to that of the *Grampus griseus* click type, and modal ICI is shorter, like delphinid species. Similarly, the majority of clusters labeled as BWG corresponded to detections of non-beaked whale clicks whose waveform seemed to be noisy or have many reverberations and thus resembled the characteristic long-duration clicks of BWG. Unfortunately, this class did not find potential BWG signals, and was instead triggered by noisy signals. For the other species of beaked whales, a significant number of false positive bins (which were not manually labeled) appeared to be correctly identified by the neural network, in particular all of the clusters categorized as Mb (**Fig 6**). A large proportion also appears to have been correctly classified as Me and Mm, and a smaller number as Zc. All of these clusters contained fewer than 20 clicks, making it difficult to manually identify the species if they were isolated, as the context of click around an acoustic event across multiple bins improves manual classification.

An ad hoc experiment revealed that adding white noise when creating a balanced dataset to improve the variability of those rare classes for training the neural network yielded comparable results when classifying the case study dataset (**S4 Table**). However, when the number of examples used for training the neural network was increased from 1000 to 5000 examples per class, the performance of the common species improved significantly with a higher precision rate (**S5 Table**), presumably because it included more examples under varying conditions and thus had higher generalization to properly classify novel data, whereas the rare classes showed no improvement.

### Case study: Comparative performance of a generalized and a targeted species classification pipeline

Using the dataset at site WC, three classification methods were compared: the generalized species classification pipeline (G), and a targeted pipeline with two different strategies to delimitate the data to the taxonomic family of beaked whales based on a hard negative filter (Th), or a moderate negative filter (Tm) (**Table 3**). Based on the manually labeled detections as true labels, both Th and Tm pipelines showed higher recall rates than the G pipeline (with average recall rates of 88%—Th, 82%—Tm, and 77%—G). However, recall rates were more similar when considering only full-misclassified bins (with average recall rates of 90%—Th, 88%—Tm, and 86%—G).

**Table 3:** Confusion matrix for all classification pipelines on the case study dataset at site WC. Values show the total number of 5-minute bins classified per class organized by class and pipeline used: a targeted species classification pipeline with a hard negative filter (Th), or a moderate negative filter (Tm), and a generalized species classification pipeline (G). Manually labeled bins are the true class. Refer to **Table 1** for abbreviation IDs. Bins with no clear assignment are indicated with the class abbreviation in italic. The total number of false negative (FN) and false positives (FP), precision and recall rates are shown per species and in parentheses when only full-misclassified bins are considered and those that are partially misclassified are excluded.

		Pipeline	Predicted Class												# FN (full bin)	% Recall (full bin)
			Mb	Zc	Mm	BWG	Md	Me	De spp	Gg	Ko spp	Pm-boat	ES ping	*No label*		
**True Class**	Mb	Th	**393**	1	6	2	-	-	12	-	-	-	-	11	32 (28)	92.5 (93.3)
		Tm	**391**	-	26	-	-	5	23	1	3	2	-	17	77 (30)	83.5 (92.9)
		G	**391**	-	23	-	-	3	27	-	4	4	-	22	83 (30)	82.5 (92.9)
	Zc	Th	-	**326**	2	4	-	2	27	7	1	1	3	5	52 (48)	86.2 (87.2)
		Tm	1	**304**	1	-	-	-	42	1	-	1	4	42	92 (70)	76.8 (81.3)
		G	2	**286**	1	-	1	-	75	5	-	4	4	36	128 (88)	69.1 (76.5)
	Mm	Th	4	2	**143**	-	-	9	4	1	2	-	-	3	25 (19)	85.1 (88.3)
		Tm	5	-	**147**	-	-	2	5	-	1	-	-	14	27 (15)	84.5 (90.7)
		G	6	-	**142**	-	-	2	13	2	1	-	-	14	38 (20)	78.9 (87.7)
	*Mm|Me*	Th	-	-	9	-	-	2	-	-	-	-	-	-	11	
		Tm	-	-	8	-	-	-	-	-	-	-	-	-	8	
		G	-	-	8	-	-	-	2	-	-	-	-	2	12	
	*No label*	Th	9	160	22	176	55	21	1147	161	4	33	47	891	2726	
		Tm	244	269	216	99	30	41	10837	1102	306	6642	225	1435	21446	
		G	218	257	205	119	28	40	10749	1071	220	7246	272	711	21136	
**# FP (full bin)**		Th	13 (9)	163 (162)	39 (37)	182	55	34	1190	169	7	34	50	910		
		Tm	250 (241)	269 (268)	251 (246)	99	30	48	10907	1104	310	6645	229	1508		
		G	226 (215)	257 (252)	237 (230)	119	29	45	10866	1078	225	7254	276	785		
**% Precision (full bin)**		Th	96.8 (97.8)	66.7 (66.8)	78.6 (79.4)											
		Tm	61.0 (61.9)	53.1 (53.2)	36.9 (37.4)											
		G	63.4 (64.5)	52.7 (53.2)	37.5 (38.2)											

**Fig 7:**
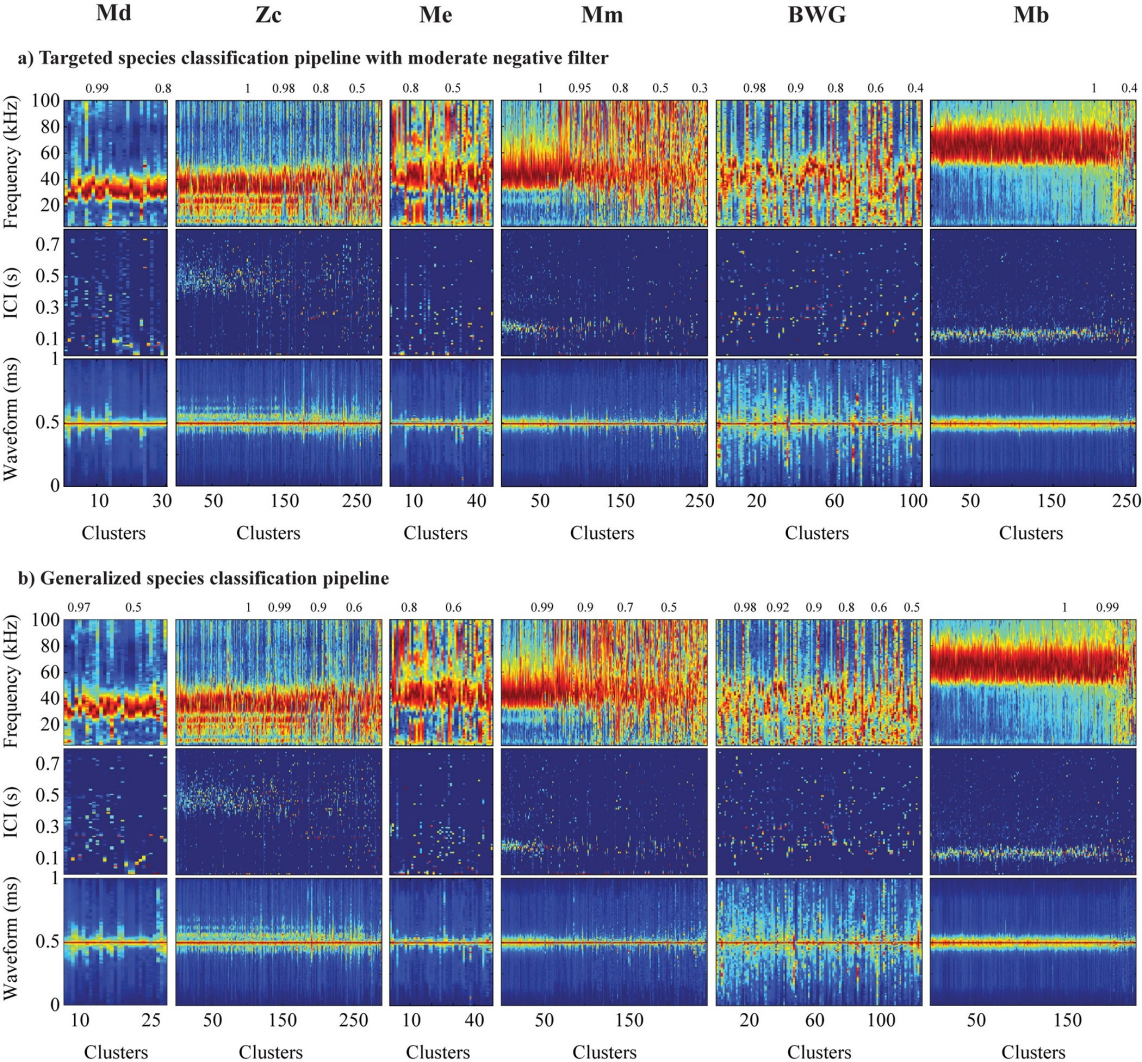
Case study dataset bins at site WC without a manual label and classified by a targeted species pipeline with a moderate negative filter and a generalized species pipeline. For each predicted beaked whale class, concatenated mean cluster spectra (top panel), concatenated cluster ICI distributions (middle panel), and concatenated mean cluster waveform envelope (bottom panel) are shown. Concatenated clusters have been sorted by site and classification prediction probability score. Color map is normalized amplitudes on a scale from 0 (dark blue) to 1 (dark red). Many of the clusters counted as misclassifications appear to have been correctly classified by the network, but were not labeled in the manual dataset. Refer to **Table 1** for abbreviation IDs, and **Fig 6** for bins without a manual label and classified by a targeted species pipeline with a hard negative filter.

Precision was low for both the Tm and G pipelines, because both methods had an abundance of possible false positive bins which were not manually labeled. Inspecting the averaged features of clusters in these bins, many clusters were correctly allocated to beaked whale species (**Fig 7**). Both methods correctly labeled at least 200 clusters as Mb, 150 clusters as Zc, and 60 clusters as Mm. These clusters originated from bins (corresponding to 160, 90 and 45 unique 5-min bins for *Ziphius cavirostris*, *Mesoplodon bidens*, and *M*. *mirus*, respectively) that were not accessible for manual classification or for automated classification using the targeted species classification with a hard negative filter, as the set of rules implemented in both methods likely removed them as failing to meet all the criteria. The remaining false positive clusters without a manual label for these classes (Mb, Zc, and Mm) were indistinguishable from averaged features and further inspection of the individual clicks would be required to make a class determination. Overall, false positive clusters assigned to the other beaked whale classes (M*d*, Me and BWG) appear to represent genuine misclassifications. Regardless, this implied that the precision rates for *Ziphius cavirostris*, *Mesoplodon bidens*, and *M*. *mirus* were underestimated, and recall rates for the Th pipeline were overestimated. Adjusting performance metrics based on the approximate number of clusters found to be correctly labeled by the Tm and G pipelines, the Th pipeline had lower recall rates than the Tm and G pipeline for *Ziphius cavirostris* (62%—Th, 83%—Tm, and 77%—G), *Mesoplodon bidens* (63%—Th, 89%—Tm, and 87%—G), and *M*. *mirus* (63%—Th, 89%—Tm, and 84%—G). Overall, the Tm pipeline had the highest recall rates for the three beaked whale species. In contrast, precision rates were potentially similar for *Mesoplodon bidens* using the different classification pipelines (97%—Th, 92%—Tm, and 96%—G) and also for *Ziphius cavirostris* (67%—Th, 79%—Tm, and 80%—G), and higher with the Th pipeline for *Mesoplodon mirus* (79%—Th, 57%—Tm, and 58%—G).

The efficiency of the three classification pipelines for processing 104GB of data at site WC was compared using a desktop computer with 32 GB of RAM and a 6-core CPU, taking all pipeline steps into account (beginning with a generic pulse detector to finally labeling clusters with the trained DNN). By far the most time-efficient of the three approaches was the Th pipeline, which only needed seven hours to classify the detections at site WC, compared to two days for the Tm pipeline, and up to 2 days and 18 hours when using the G pipeline.

## Discussion

Rare scenarios, such as those involving rare species or acoustic ephemeral events, are more likely to be missed or misclassified than common species or longer-lasting events because they present fewer opportunities for data acquisition and limited information to capture the variability. Here we illustrated the difficulties inherent in recognizing such occurrences when using the same automated techniques to classify the sounds of many species of toothed whales simultaneously. Automated classification of all possible clicks is difficult, and often analysts cannot determine whether weak or isolated signals are clicks. Even strong clicks can be difficult to find with certainty when they are isolated because pulse signals have limited information [31]. Such manual classification decisions often rely on contextual information like clicking rate and proximity of similar and high-amplitude signals, which ease the classification of bins with varying numbers of clicks. However, relying too much on context when constructing automatic classifiers can negatively affect their performance on rare signal events [33, 34]. Recent studies have used both deep learning [33–35] and random forest [29] methods for automated classification of beaked whale echolocation clicks. While both approaches are effective, deep learning demonstrates higher and more robust classification performance [29, 33–35]. Our study supports these findings, showing high classification performance comparable with results of a deep learning method previously applied to the western North Atlantic [34], with both classifiers achieving precision and recall rates above 98% for beaked whale species in the test datasets. However, these studies also highlight the challenges faced by generalized species classifiers in accurately classifying rare or ephemeral events in datasets beyond the test performance phase [33, 34]. This underscores the need for methodological improvements tailored toward identification of these events.

We described an automated approach to delimiting signals to the taxonomic family of beaked whales by removing dominant non-beaked whale signals, which improved the efficiency of classifying beaked whale species and lowered misclassification detection rates. The generalized classifier implemented in the case study example was implemented to be comparable with prior deep-learning-based methods [33–35] as a baseline model, to facilitate comparison with our proposed targeted species classification pipeline. A random forest approach utilizing a hard negative filter to eliminate non-beaked whale species based on frequency sweep rate of detected clicks [29] has also been proposed, with slightly poorer performance than the baseline model. Our comparison results shows that when non-beaked whale dominant clicks were not removed prior to clustering, a higher incidence of partial misclassification of time bins occurred. In these instances, several clicks of beaked whales were incorrectly classified to another class within a time bin, leading to higher false positive click detections, despite some clicks within the same window being correctly classified. The targeted species classification pipeline with a hard negative filter eliminated most non-beaked whale dominant signals and of those remaining signals high classification accuracy to species level was reached, but the fixed set of rules also eliminated time bins with beaked whale presence. This is likely to increase the false negative rates as many detections in time windows did not meet all of the criteria. This highlights the primary challenge with delimitation, care must be taken to define partitioning criteria which effectively remove the bulk of non-target signals, without significant removal of target signals. This requires that the group of signals have a robust set of shared features. A delimitation phase can be thought of as the application of a weak classifier, which is then followed by a stronger DNN classifier in this case. The targeted species classification pipeline with a moderate negative filter only used averaged feature characteristics describing spectral shape, click duration, and consistent clicking rate, and was able to partially remove non-beaked whale dominant signals providing a balanced performance between false negative and false positive detections. All methods evaluated here can yield comparable results when the presence of beaked whale species is assessed at coarse time scale (e.g. hourly, daily). However, if finer resolution to the click or minute level is needed, such as for studies to understand beaked whale responses to noise exposure, a machine learning pipeline with a targeted species classification can achieve lower misclassification rates while also being more computationally efficient.

The application of a delimitation phase to the taxonomic family of beaked whales before unsupervised clustering allowed for the grouping of signals of smaller and varying numbers, and improved classification of beaked whale ephemeral events from long-term passive acoustic monitoring recordings as shown by comparing the classifications made by a manual review with a targeted species pipeline with a hard negative filter. The trained DNN had the ability to retrieve a high number of bins with beaked whale presence and showed high performance in classifying bins with a limited number of clicks. Our study shows that specifying the minimum number of clicks per time bin necessary for unsupervised clustering should be done with care, as doing so can result in high misclassification rates. When time intervals did not reach the minimum number of detections per bin, unsupervised clustering was not applied and all signals were averaged to compute the features for classification. This can result in misclassifications because all signals are grouped when they are insufficient for clustering, particularly if the few signals present correspond with more than one species and the most numerous clicks would obscure the rare signals. An alternative could be to classify signals independently when not reaching the clustering settings, though this approach may as well result in high misclassification because relevant features such as the modal ICI could not be computed. A better understanding of the distribution of the number of signals per time interval of the species of interest would be recommended when using unsupervised clustering methods.

False positive classifications obtained in the novel dataset for the rare classes warrant consideration, setting up a balanced dataset with extremely rare species may not result in increased classification performance. Training a DNN with an unbalanced dataset can lead to the classifier learning that the common classes are more likely, causing it to categorize more signals as belonging to the common classes. However, creating a balanced dataset for training can lead to a brittle classifier as common classes are subsampled and samples with varied conditions of background noise and variability within these broadband directional clicks are reduced. We have shown that increasing the number of samples for training increased the precision of the classifier, although the performance for the rarest classes, even when adding noise, did not improve as they are likely to be over-fitted because the classifier did not have enough examples to learn the species’ distinctive features. This appears to happen despite signals like the BWG type, which have very distinct features.

Although our classifier is focused on a particular taxonomic group, we found that the inclusion of clearly defined non-target categories was necessary. DNNs assign the most probable label, regardless of the probability of that label, therefore if no good match exists, a poor one will be selected resulting in misclassifications. In this study, significant number of false positive clusters appeared to be detections of a click type not represented in the training dataset. This click type shared similarities with the clicks of *Grampus griseus*, but its lower frequency peaks were not present. This click type was recently named UD36 [34] and, due to its similarities in spectral pattern, click rate, and temporal overlap with *Grampus griseus* signals, the UD36 click type was proposed as a potential alternative *Grampus griseus* signal or other species that often co-occur with this species. Including this click type in the training dataset would potentially reduce spurious assignments of the type to various beaked whale classes.

To perform effectively and comparably across different regions, training sets must be developed to reflect the full variability of each class over those regions. Previous research has found subtle differences in the spectral shape of *Grampus griseus* clicks between regions and ocean basins [50]. Intra-specific variability is less understood for beaked whales, and only differences in the spectral shape and click rates have been documented for *Mesoplodon densirostris* [17]. Here, we demonstrate that the click characteristics of *M*. *densirostris* differ considerably across regions and ocean basins, as measured by clicking rates, spectral patterns, click duration, but that the other North Atlantic beaked whale species did not. The Md click type from the training dataset had a moderate number of samples and contained intraspecific variability, and most likely contributed to a poorer performance than the other beaked whale species. Larger training sample sizes are needed in such cases to capture the variability of these species across regions and achieve the desired classification performance.

Site-specific noise and instrumentation differences may further confuse classifiers [51], and could partially account for misclassifications between subtly different types which are not detected within the same dataset sites, as in the case of *Mesoplodon mirus* and *M*. *europaeus*. In these cases, a classifier may mistakenly learn to use non-species-specific features for classification during training. Inclusion of additional strategies, such as domain adaptation [52], in the pipeline may be needed to improve the robustness of these methods across locations and sensor types. Because of the inherent heterogeneity of the beaked whale species’ occurrence, the datasets will likely show a long-tailed distribution, with some classes having many more samples than others. A possible strategy may be to test recently developed techniques to handle long-tailed learning [53], some of which reduce the bias in the feature space and decision boundaries between common and rare classes.

The application of this system in the case study sites where it was not trained revealed variable numbers of false negative and false positives, with the latter being largely influenced by the presence of non-target species not included in the training dataset. This scenario highlights the challenges of applying a trained classifier to other regions with different species not accounted for during training. To ensure high performance, it is advisable to train the deep neural network with signals that more accurately reflect the true distribution of classes in the region (e.g., ocean basin). These issues are particularly important for density estimation methods that rely on being able to adequately characterize the classifier’s performance [36]. Classifier performance metrics can be reported per site, but quality control using machine-aided analysis, such as *DetEdit* [42] which allows rapid estimation of a classifier’s false positive rate over a systematic random sample, remains particularly important in these cases.

Applying the trained neural network to new data can produce additional samples of each species, which can then be used to retrain the classifier and further improve its accuracy. As a result, we hope to ensure the continued availability of data and promote more representation of rare species by making the training data public.

## Conclusions

Findings from this study can aid in determining better practices for the design and use of clustering approaches with trained deep neural networks. We have shown that the detection and classification process using machine learning is more efficient and can lower the overall rate of missed detections of ephemeral acoustic events when the pipeline is tailored to focus on the taxonomic family of beaked whales. A delimitation phase to remove common non-target species before unsupervised clustering has facilitated the grouping of signals of varying numbers, and performed well in classifying clusters with a smaller number of clicks. The results demonstrate that the minimal number of clicks per time bin necessary for unsupervised clustering, as well as a representative training set of all non-target species, should be carefully chosen to avoid high misclassification rates. We have shown that there are a variety of rare cases to consider when classifying passive acoustic data, and that the approach presented improved the detection of ephemeral events, but rare species, or species with intraspecific variability in their click characteristic features, remain a challenge in machine learning classification, and we expect that this study will not only improve understanding but also aid in identifying potential innovation directions in rare class learning.

## Supporting information

S1 Table Deployment summary for all regions and sites with HARP recordings. (PDF)

S2 Table Detection summary for the manual and targeted species classification pipeline from the case study dataset. (PDF)

S3 Table Confusion matrix for the targeted species classification pipeline with a hard negative filter per site. (PDF)

S4 Table Confusion matrix for the targeted species classification pipeline with a hard negative on the case study dataset with training sample size 1000 and added noise to increase variability. (PDF)

S5 Table Confusion matrix for the targeted species classification pipeline with a hard negative on the case study dataset with training sample size 5000 and added noise to increase variability. (PDF)

S1 Fig Blainville’s beaked whale signal class organized by region formed using unsupervised clustering at a 5-min bin level based on spectra, inter-click-interval (ICI), and waveform envelope. (PDF)

S2 Fig Clusters misclassified by the deep neural network in the balanced test set. (PDF)

S1 File (DOCX)

## References

[1] Dalebout ML, Mead JG, Baker CS, Baker AN, van Helden AL. A new species of beaked whale *Mesoplodon perrini* sp. N. (Cetacea: Ziphiidae) discovered through phylogenetic analyses of mitochondrial DNA sequences. Mar Mammal Sci. 2002;18: 577–608. 10.1111/j.1748-7692.2002.tb01061.x

[2] Carroll EL, McGowen MR, McCarthy ML, Marx FG, Aguilar N, Dalebout ML, et al. Speciation in the deep: genomics and morphology reveal a new species of beaked whale *Mesoplodon eueu*. Proc R Soc B Biol Sci. 2021;288: 20211213. doi: 10.1098/rspb.2021.1213 34702078 PMC8548795

[3] Brownell RL Jr., Kasuya T. Sato’s beaked whale: A new cetacean species discovered around Japan. Mar Mammal Sci. 2021;37: 768–771. 10.1111/mms.12810

[4] Committee on Taxonomy. List of marine mammal species and subspecies. Society for Marine Mammology. Available: www.marinemammalscience.org

[5] IUCN. 2022. The IUCN Red List of Threatened Species. Version 2022–2. [cited 6 Jul 2023]. Available: https://www.iucnredlist.org

[6] Cox TM, Ragen TJ, Read AJ, Vos E, Baird RW, Balcomb KC III, et al. Understanding the impacts of anthropogenic sound on beaked whales. Journal of Cetacean Research and Management. 2006. pp. 177–187. doi: 10.1109/LPT.2009.2020494

[7] Hooker SK, De Soto NA, Baird RW, Carroll EL, Claridge D, Feyrer L, et al. Future directions in research on beaked whales. Front Mar Sci. 2019;6. doi: 10.3389/fmars.2018.00514

[8] MacLeod CD, Santos MB, Pierce GJ. Review of data on diets of beaked whales: Evidence of niche separation and geographic segregation. J Mar Biol Assoc United Kingdom. 2003;83: 651–665. doi: 10.1017/S0025315403007616h

[9] Tyack PL, Johnson M, Soto NA, Sturlese A, Madsen PT. Extreme diving of beaked whales. J Exp Biol. 2006;209: 4238–53. doi: 10.1242/jeb.02505 17050839

[10] Barlow J. Inferring trackline detection probabilities, g(0), for cetaceans from apparent densities in different survey conditions. Mar Mammal Sci. 2015;31: 923–943. 10.1111/mms.12205

[11] Johnson M, Madsen PT, Zimmer WMX, de Soto NA, Tyack PL. Beaked whales echolocate on prey. Proc Biol Sci. 2004;271: S383–S386. doi: 10.1098/rsbl.2004.0208 15801582 PMC1810096

[12] Zimmer WMX, Johnson MP, Madsen PT, Tyack PL. Echolocation clicks of free-ranging Cuvier’s beaked whales (*Ziphius cavirostris*). J Acoust Soc Am. 2005;117: 3919–3927. doi: 10.1121/1.1910225 16018493

[13] Baumann-Pickering S, McDonald MA, Simonis AE, Solsona-Berga A, Merkens KPB, Oleson EM, et al. Species-specific beaked whale echolocation signals. J Acoust Soc Am. 2013;134: 2293–301. Available: http://www.ncbi.nlm.nih.gov/pubmed/23967959 doi: 10.1121/1.4817832 23967959

[14] Baumann-Pickering S, Roch MA, Brownell RL Jr, Simonis AE, McDonald MA, Solsona-Berga A, et al. Spatio-temporal patterns of beaked whale echolocation signals in the North Pacific. Fahlman A, editor. PLoS One. 2014;9: e86072. doi: 10.1371/journal.pone.0086072 24465877 PMC3899217

[15] Baumann-Pickering S, Wiggins SM, Roth EH, Roch MA, Schnitzler H-U, Hildebrand JA. Echolocation signals of a beaked whale at Palmyra Atoll. J Acoust Soc Am. 2010;127: 3790–9. doi: 10.1121/1.3409478 20550277

[16] Stanistreet JE, Nowacek DP, Baumann-Pickering S, Bell JT, Cholewiak DM, Hildebrand JA, et al. Using passive acoustic monitoring to document the distribution of beaked whale species in the western North Atlantic Ocean. Can J Fish Aquat Sci. 2017;74: 2098–2109. doi: 10.1139/cjfas-2016-0503

[17] Baumann‐Pickering S, Trickey JS, Solsona‐Berga A, Rice A, Oleson EM, Hildebrand JA, et al. Geographic differences in Blainville’s beaked whale (*Mesoplodon densirostris*) echolocation clicks. Divers Distrib. 2023;00: 1–14. doi: 10.1111/ddi.13673

[18] Manzano-Roth R, Henderson EE, Alongi GC, Martin CR, Martin SW, Matsuyama B. Dive characteristics of Cross Seamount beaked whales from long-term passive acoustic monitoring at the Pacific Missile Range Facility, Kauaʻi. Mar Mammal Sci. 2023;39: 22–41. doi: 10.1111/mms.12959

[19] Hildebrand JA, Baumann-Pickering S, Frasier KE, Trickey JS, Merkens KP, Wiggins SM, et al. Passive acoustic monitoring of beaked whale densities in the Gulf of Mexico. Sci Rep. 2015;5: 16343. doi: 10.1038/srep16343 26559743 PMC4642294

[20] Henderson EE, Martin SW, Manzano-Roth R, Matsuyama BM. Occurrence and habitat use of foraging Blainville’s beaked whales (*Mesoplodon densirostris*) on a U.S. Navy range in Hawaii. Aquat Mamm. 2016;42: 549–562. doi: 10.1578/AM.42.4.2016.549

[21] Gillespie D, Gordon J, Mchugh R, Mellinger DK, Redmond P, Thode A, et al. PAMGUARD: open source software for real-time acoustic detection and localisation of cetaceans. Proc Inst Acoust. 2008;30: 9. Available: www.pamguard.org

[22] Klinck H, Mellinger DK, Klinck K, Bogue NM, Luby JC, Jump WA., et al. Near-real-time acoustic monitoring of beaked whales and other cetaceans using a Seaglider. PLoS One. 2012;7: 1–8. doi: 10.1371/journal.pone.0036128 22629309 PMC3356361

[23] Matsumoto H, Jones C, Klinck H, Mellinger DK, Dziak RP, Meinig C. Tracking beaked whales with a passive acoustic profiler float. J Acoust Soc Am. 2013;133: 731–740. doi: 10.1121/1.4773260 23363092

[24] Yack TM, Barlow J, Calambokidis J, Southall B, Coates S. Passive acoustic monitoring using a towed hydrophone array results in identification of a previously unknown beaked whale habitat. J Acoust Soc Am. 2013;134: 2589–2595. doi: 10.1121/1.4816585 23968056

[25] Yack TM, Barlow J, Roch MA., Klinck H, Martin S, Mellinger DK, et al. Comparison of beaked whale detection algorithms. Appl Acoust. 2010;71: 1043–1049. doi: 10.1016/j.apacoust.2010.04.010

[26] Roch MA, Soldevilla MS, Hoenigman R, Wiggins SM, Hildebrand JA. Comparison of machine learning techniques for the classification of echolocation clicks from three species of odontocetes. Can Acoust. 2008;36: 41–47.

[27] Jarvis S, DiMarzio N, Morrissey R, Moretti D. A novel multi-class support vector machine classifier for automated classification of beaked whales and other small odontocetes. Can Acoust. 2008;36. Available: http://jcaa.caa-aca.ca/index.php/jcaa/article/view/1988

[28] Luo W, Yang W, Zhang Y. Convolutional neural network for detecting odontocete echolocation clicks. J Acoust Soc Am. 2019;145: EL7–EL12. doi: 10.1121/1.5085647 30710948

[29] Rankin S, Sakai T, Archer FI, Barlow J, Cholewiak D, DeAngelis AI, et al. Open-source machine learning BANTER acoustic classification of beaked whale echolocation pulses. Ecol Inform. 2024;80: 102511. doi: 10.1016/j.ecoinf.2024.102511

[30] LeBien JG, Ioup JW. Species-level classification of beaked whale echolocation signals detected in the northern Gulf of Mexico. J Acoust Soc Am. 2018;144: 387–396. doi: 10.1121/1.5047435 30075691

[31] Frasier KE, Roch MA, Soldevilla MS, Wiggins SM, Garrison LP, Hildebrand JA. Automated classification of dolphin echolocation click types from the Gulf of Mexico. PLOS Comput Biol. 2017;13: e1005823. doi: 10.1371/journal.pcbi.1005823 29216184 PMC5720518

[32] Li K, Sidorovskaia NA, Tiemann CO. Model-based unsupervised clustering for distinguishing Cuvier’s and Gervais’ beaked whales in acoustic data. Ecol Inform. 2020;58: 101094. doi: 10.1016/j.ecoinf.2020.101094

[33] Frasier KE. A machine learning pipeline for classification of cetacean echolocation clicks in large underwater acoustic datasets. PLoS Comput Biol. 2021;17: 1–26. doi: 10.1371/journal.pcbi.1009613 34860825 PMC8673644

[34] Cohen RE, Frasier KE, Baumann-Pickering S, Wiggins SM, Rafter MA, Baggett LM, et al. Identification of western North Atlantic odontocete echolocation click types using machine learning and spatiotemporal correlates. PLoS One. 2022;17: 1–37. doi: 10.1371/journal.pone.0264988 35324943 PMC8946748

[35] Ziegenhorn MA, Frasier KE, Hildebrand JA, Oleson EM, Baird RW, Wiggins SM, et al. Discriminating and classifying odontocete echolocation clicks in the Hawaiian Islands using machine learning methods. PLoS One. 2022;17: 1–24. doi: 10.1371/journal.pone.0266424 35413068 PMC9004765

[36] Marques TA, Thomas L, Ward J, DiMarzio N, Tyack PL. Estimating cetacean population density using fixed passive acoustic sensors: an example with Blainville’s beaked whales. J Acoust Soc Am. 2009;125: 1982–94. doi: 10.1121/1.3089590 19354374

[37] Deruiter SL, Southall BL, Calambokidis J, Zimmer WMX, Sadykova D, Falcone EA, et al. First direct measurements of behavioural responses by Cuvier’s beaked whales to mid-frequency active sonar. Biol Lett. 2013;9: 0–4. doi: 10.1098/rsbl.2013.0223 23825085 PMC3730631

[38] Cholewiak D, DeAngelis AI, Palka D, Corkeron PJ, Van Parijs SM. Beaked whales demonstrate a marked acoustic response to the use of shipboard echosounders. R Soc Open Sci. 2017;4. doi: 10.1098/rsos.170940 29308236 PMC5750003

[39] Aguilar Soto N, Johnson M, Madsen PT, Tyack PL, Bocconcelli A, Fabrizio Borsani J. Does intense ship noise disrupt foraging in deep-diving cuvier’s beaked whales (*Ziphius cavirostris*)? Mar Mammal Sci. 2006;22: 690–699. doi: 10.1111/j.1748-7692.2006.00044.x

[40] Marine BioAcoustics Research Collaborative. Triton software package. 2024. Available: https://github.com/MarineBioAcousticsRC/Triton. doi: 10.5281/zenodo.10963175

[41] Wiggins SM, Hildebrand JA. High-frequency Acoustic Recording Package (HARP) for broad-band, long-term marine mammal monitoring. International Symposium on Underwater Technology 2007 and International Workshop on Scientific Use of Submarine Cables & Related Technologies 2007. Institute of Electrical and Electrongics Engineers, Tokyo, Japan; 2007. pp. 551–557.

[42] Solsona-Berga A, Frasier KE, Baumann-Pickering S, Wiggins SM, Hildebrand JA. DetEdit: A graphical user interface for annotating and editing events detected in long-term acoustic monitoring data. Schneidman-Duhovny D, editor. PLOS Comput Biol. 2020;16: e1007598. doi: 10.1371/journal.pcbi.1007598 31929520 PMC6980688

[43] Székely GJ, Rizzo ML, Bakirov NK. Measuring and testing dependence by correlation of distances. Ann Stat. 2007;35: 2769–2794. doi: 10.1214/009053607000000505

[44] Biemann C. Chinese whispers—An efficient graph clustering algorithm and its application to natural language processing problems. Proc TextGraphs 1st Work Graph-Based Methods Nat Lang Process. 2006; 73–80.

[45] Fred ALN, Jain AK. Combining multiple clusterings using evidence accumulation. IEEE Trans Pattern Anal Mach Intell. 2005;27: 835–850. doi: 10.1109/TPAMI.2005.113 15943417

[46] Maas AL, Hannun AY, Ng AY. Rectifier nonlinearities improve neural network acoustic models. ICML Work Deep Learn Audio, Speech Lang Process. 2013;28.

[47] Bridle JS. Probabilistic interpretation of feedforward classification network outputs, with relationships to statistical pattern recognition. In: Soulié FF, Hérault J, editors. Neurocomputing. Berlin, Heidelberg: Springer Berlin Heidelberg; 1990. pp. 227–236.

[48] Hildebrand JA, Frasier KE, Helble TA, Roch MA. Performance metrics for marine mammal signal detection and classification. J Acoust Soc Am. 2022;151: 414–427. doi: 10.1121/10.0009270 35105012

[49] Wimmer T, Whitehead H. Movements and distribution of northern bottlenose whales, *Hyperoodon ampullatus*, on the Scotian Slope and in adjacent waters. Can J Zool. 2004;82: 1782–1794. doi: 10.1139/Z04-168

[50] Soldevilla MS, Baumann-Pickering S, Cholewiak D, Hodge LEW, Oleson EM, Rankin S. Geographic variation in Risso’s dolphin echolocation click spectra. J Acoust Soc Am. 2017;142: 599–617. doi: 10.1121/1.4996002 28863585

[51] Roch MA, Lindeneau S, Aurora GS, Frasier KE, Hildebrand JA, Glotin H, et al. Using context to train time-domain echolocation click detectors. J Acoust Soc Am. 2021;149: 3301–3310. doi: 10.1121/10.0004992 34241092

[52] Olvera M, Vincent E, Gasso G. On The impact of normalization strategies in unsupervised adversarial domain adaptation for acoustic scene classification. ICASSP 2022–2022 IEEE International Conference on Acoustics, Speech and Signal Processing (ICASSP). 2022. pp. 631–635. doi: 10.1109/ICASSP43922.2022.9747540

[53] Zhang Y, Kang B, Hooi B, Yan S, Feng J. Deep long-tailed learning: a survey. IEEE Trans Pattern Anal Mach Intell. 2023;PP: 1–20. doi: 10.1109/TPAMI.2023.3268118 37074896

