# Supplementary material for: Machine learning with taxonomic family delimitation aids in the classification of ephemeral beaked whale events in passive acoustic monitoring
Source: PLoS One. 2024 Jun 4;19(6):e0304744. doi: 10.1371/journal.pone.0304744 (PMC11149863; doi:10.1371/journal.pone.0304744)
Supplement: S1 Table — (PDF) [file pone.0304744.s001.pdf]

Supplementary Material

Table S1. Deployment summary for all regions and sites with HARP recordings. Number of deployments per site, location, bottom depth, start and end date of effort, total days with recording, hydrophone crossover frequency between low- and high-frequency band sensor elements, data use for training, and case study (CS). Region abbreviations: Western North Atlantic – WAT and Gulf of Mexico – GOM.

Region	Site	Deployment	Latitude (°N)	Longitude (°W)	Depth (m)	Start date (UTC)	End date (UTC)	Recording days	Crossover freq. (kHz)	Data usage
**WAT**	HZ – Heezen Canyon	1	41-03.715	66-21.092	845	06/27/2015	03/25/2016	271	25	Training
		2	41-03.710	66-21.095	845	07/01/2016	08/31/2016	62	--	CS
	OC – Oceanographer Canyon	1	40-15.799	67-59.174	450	07/01/2016	08/31/2016	62	--	CS
	BR – Bear Seamount	1	40-01.967	67-59.301	2085	07/25/2018	08/17/2018	23	--	Training
	NC – Nantucket Canyon	1	39-49.949	69-58.928	977	04/27/2015	09/18/2015	145	25	Training
		2	39-49.943	69-58.926	977	07/01/2016	08/31/2016	62	--	CS
	BC – Babylon Canyon	1	39-11.463	72-13.722	1000	07/01/2016	08/31/2016	62	--	CS
	WC – Wilmington Canyon	1	38-22.449	73-22.241	1000	07/01/2016	08/31/2016	62	--	CS
	NFC – Norfolk Canyon	2	37-09.991	74-27.996	968	04/30/2016	06/28/2017	424	--	Training
		3	37-10.044	74-27.980	950	06/30/2017	06/02/2018	337	2	Training
	HAT – Hatteras	1	35-35.011	74-44.584	1200	07/01/2016	08/31/2016	62	--	Training
	ONB – Onslow Bay	1	33-46.676	75-55.585	952	08/19/2011	12/01/2011	105	2	Training
		2	33-47.200	75-55.750	914	07/14/2012	10/02/2012	81	2	Training
	GS – Gulf Stream	1	33-39.938	76-00.083	953	07/01/2016	08/31/2016	62	--	CS
	BP – Blake Plateau	1	32-06.362	77-05.659	945	07/01/2016	08/31/2016	62	--	CS
	BM – Bermuda	1	31-55.575	65-12.900	713	06/10/2013	03/11/2014	274	25	Training
		2	31-55.415	65-12.113	732	03/13/2014	07/21/2014	131	25	Training
		3	31-55.415	65-12.113	732	12/17/2014	10/02/2015	289	25	Training
	BS – Blake Spur	1	30-35.027	77-23.443	1005	04/27/2016	06/26/2017	425	--	Training
	JAX – Jacksonville	1	30-09.036	79-46.203	800	08/23/2014	05/29/2015	279	10	Training
		2	30-09.135	79-46.236	740	06/27/2018	06/15/2019	354	--	Training
**GOM**	MC – Mississippi Canyon	1	28-50.797	88-27.991	980	09/22/2011	02/21/2012	152	2	Training
		2	28-50.853	88-28.041	980	02/28/2012	12/11/2012	288	2	Training
	GC – Green Canyon	1	27-33.470	91-10.010	1115	07/15/2010	10/11/2010	88	2	Training
		2	27-33.466	91-10.014	1160	11/08/2010	02/02/2011	86	2	Training
		3	27-33.424	91-10.073	1100	03/23/2011	08/08/2011	138	2	Training
		4	27-33.426	91-10.060	1100	09/23/2011	02/17/2012	118	2	Training
		5	27-33.440	91-10.562	1100	02/28/2012	12/12/2012	289	2	Training
	DT – Dry Tortugas	1	25-31.911	84-38.251	1320	08/09/2010	10/26/2010	79	2	Training
		2	25-31.911	84-38.251	1320	03/04/2011	06/24/2011	111	2	Training
		3	25-32.360	84-37.743	1210	06/22/2016	07/18/2017	392	--	Training
	HH – Howell Hook	1	25-01.702	84-23.769	1050	05/27/2012	12/06/2012	194	2	Training
