# Supplementary material for: Machine learning with taxonomic family delimitation aids in the classification of ephemeral beaked whale events in passive acoustic monitoring
Source: PLoS One. 2024 Jun 4;19(6):e0304744. doi: 10.1371/journal.pone.0304744 (PMC11149863; doi:10.1371/journal.pone.0304744)
Supplement: S2 Table — (PDF) [file pone.0304744.s002.pdf]

Supplementary Material

Table S2. Detection summary for the manual and targeted species classification pipeline from the case study dataset. Total number of 5-minute bins with species presence across all sites between July and August 2016. The targeted species classification pipeline implemented a hard negative filter.

	HZ	OC	NC	BC	WC	GS	BP	All Sites
**Manual classification:**								
BWG	0	0	0	0	0	0	0	0
Mb	297	45	25	59	421	0	0	847
Md	0	0	0	0	0	0	6	6
Me	0	0	0	1	0	1033	2157	3191
Mm	0	0	94	90	162	0	0	346
Zc	786	3	24	141	374	6	0	1334
**Neural network classification:**								
BWG	113	350	1893	119	182	59	70	2786
Mb	280	36	13	59	404	0	6	798
Md	82	214	291	68	55	5	9	724
Me	48	32	69	40	33	864	1738	2824
Mm	45	93	326	153	179	168	675	1639
Zc	828	100	157	200	489	26	48	1848
