# Supplementary material for: Machine learning with taxonomic family delimitation aids in the classification of ephemeral beaked whale events in passive acoustic monitoring
Source: PLoS One. 2024 Jun 4;19(6):e0304744. doi: 10.1371/journal.pone.0304744 (PMC11149863; doi:10.1371/journal.pone.0304744)
Supplement: S3 Table — (PDF) [file pone.0304744.s003.pdf]

Supplementary Material

Table S3. Confusion matrix for the targeted species classification pipeline with a hard negative filter per site. Values indicate the total number of 5-minute bins classified. Refer to **Table 1** for abbreviation IDs. Bins with no clear assignment are indicated with the class abbreviation in italic.

a) Heezen Canyon (HZ)

	Site HZ	Predicted Class													
		Mb	Zc	BWG	Md	Me	Mm	De spp	Gg	Ko spp	Pm-boat	ES ping	*No label*	**N missed**	**Recall (%)**
**True Class**	Mb	**274**			2		4	8		2	1		8	25	91.6
	Zc	1	**713**	6	3	9	10	31	10				5	75	90.5
	Mm–Me						4							4	
	No label	5	115	107	77	39	29	597	441	6	4	4	698	2122	
	**N false alarm**	6	115	113	82	48	47	636	451	8	5	4	711		
	**Precision (%)**	97.9	86.1												

b) Oceanographic Canyon (OC)

	Site OC	Predicted Class													
		Mb	Zc	BWG	Md	Me	Mm	De spp	Gg	Ko spp	Pm-boat	ES ping	*No label*	**N missed**	**Recall (%)**
**True Class**	Mb	**35**		2			3	3	1				1	10	77.8
	Zc		**1**			1		1						2	33.3
	Mm–Me					1	1							2	
	likely Md							1						1	
	No label	1	99	348	214	30	89	1133	495	8	10	7	1331	3765	
	**N false alarm**	1	99	350	214	32	93	1138	496	8	10	7	1332		
	**Precision (%)**	97.2	1.0												

c) Nantucket Canyon (NC)

	Site NC	Predicted Class													
		Mm	Zc	Mb	BWG	Md	Me	De spp	Gg	Ko spp	Pm-boat	ES ping	*No label*	**N missed**	**Recall (%)**
**True Class**	Mm	**88**	1		3			1		1			1	7	92.6
	Zc	2	**20**		3	1								6	76.9
	Mb	1		**9**	2			2			1		10	16	36.0
	Mm–Me	11	1		2			1						15	
	likely Md									1				1	
	No label	225	135	4	1884	290	69	843	630	50	166	37	998	5331	
	**N false alarm**	239	137	4	1894	291	69	847	630	52	167	37	1009		
	**Precision (%)**	26.9	12.7	69.2											

d) Babylon Canyon (BC)

Site BC	Me	Mb	Mm	Zc	BWG	Md	De spp	Gg	Ko spp	Pm-boat	ES ping	*No label*	**N missed**	**Recall (%)**
	**Predicted Class**													
**True Class** Me	**1**												0	100.0
Mb	1	**55**					2		1				4	93.2
Mm	2		**84**	3	2		4					1	12	87.5
Zc			5	**125**	7	2	2	3				1	20	86.2
Mm–Me	2		5				1						8	
No label	34	4	61	74	111	67	468	364	12	4	1	390	1590	
**N false alarm**	39	4	71	77	120	69	477	367	13	4	1	392		
**Precision (%)**	2.5	93.2	54.2	61.9										

e) Wilmington Canyon (WC)

Site WC	Mb	Zc	Mm	BWG	Md	Me	De spp	Gg	Ko spp	Pm-boat	ES ping	*No label*	**N missed**	**Recall (%)**
	**Predicted Class**													
**True Class** Mb	**393**	1	6	2			12					11	32	92.5
Zc		**326**	2	4		2	27	7	1	1	3	5	52	86.2
Mm	4	2	**143**			9	4	1	2			3	25	85.1
Mm–Me			9			2							11	
No label	9	160	22	176	55	21	1147	161	4	33	47	891	2726	
**N false alarm**	13	163	39	182	55	34	1190	169	7	34	50	910		
**Precision (%)**	96.8	66.7	78.6											

f) Gulf Stream (GS)

Site GS	Me	Zc	BWG	Mb	Md	Mm	De spp	Gg	Ko spp	Pm-boat	ES ping	*No label*	**N missed**	**Recall (%)**
	**Predicted Class**													
**True Class** Me	**848**	11	19			150	9		2				191	81.6
Zc	1	**3**				1						1	3	50.0
Mm – Me			1			2							3	
likely Md			1										1	
likely Me			1										1	
No label	15	12	38		5	20	60	25	15	5	1	54	250	
**N false alarm**	16	23	60	0	5	173	69	25	17	5	1	55		
**Precision (%)**	98.1	11.5												

g) Blake Plateau (BP)

	Site BP	Predicted Class													
		Me	Md	BWG	Mb	Mm	Zc	De spp	Gg	Ko spp	Pm-boat	ES ping	*No label*	**N missed**	**Recall (%)**
**True Class**	Me	**1714**	4	63	3	336	40	23	3	5		1		478	78.2
	Md	1	**2**	1				3						5	28.6
	Md – Me	1												1	
	No label	23	5	7	5	363	9		79	70			410	971	
	**N false alarm**	25	9	71	8	699	49	26	82	75	0	1	410		
	**Precision (%)**	98.6	100												

h) Wilmington Canyon (WC)

	Site WC	Predicted Class													
		Mb	Zc	Mm	BWG	Md	Me	De spp	Gg	Ko spp	Pm-boat	ES ping	*No label*	**N missed**	**Recall (%)**
**True Class**	Mb	**391**	1	6	2			12					11	32	92.5
	Zc		**326**	2	4		2	27	7	1	1	3	5	52	86.2
	Mm	4	2	**143**			9	4	1	2			3	25	85.1
	Mm–Me			9			2							11	
	No label	9	160	22	176	55	21	1147	161	4	33	47	891	2726	
	**N false alarm**	13	163	39	182	55	34	1190	169	7	34	50	910		
	**Precision (%)**	96.1	66.7	78.6											
