# Supplementary material for: Machine learning with taxonomic family delimitation aids in the classification of ephemeral beaked whale events in passive acoustic monitoring
Source: PLoS One. 2024 Jun 4;19(6):e0304744. doi: 10.1371/journal.pone.0304744 (PMC11149863; doi:10.1371/journal.pone.0304744)
Supplement: S4 Table — (PDF) [file pone.0304744.s004.pdf]

Supplementary Material

Table S4. Confusion matrix for the targeted species classification pipeline with a hard negative filter on the case study dataset with training sample size 1000 and added noise to increase variability. Values indicate the total number of 5-minute bins classified. Refer to **Table 1** for abbreviation IDs. Bins with no clear assignment are indicated with the class abbreviation in italic.

		Mb	Zc	Mm	Me	Md	BWG	De spp	Gg	Ko spp	Pm-boat	ES ping	*No label*	**N missed**	**Recall (%)**
**True Class**	Mb	**776**	3	5		1	14	20		2	1		30	76	91.1
	Zc	1	**1206**	10	19	1	20	65	8	1		2	12	139	89.7
	Mm	4	10	**309**	12		10	6		2			5	49	86.3
	Me	9	66	411	**2562**	3	131	37	4	7				668	79.3
	Md				1	**2**	1	3						5	28.6
	Mm – Me			23	9		5	5	1					43	
	Md – Me				1									1	
	likely Me							1						1	
	likely Md							3						3	
	No label	47	1067	647	222	569	2941	5193	876	120	257	74	4772	16785	
	**N false alarm**	61	1146	1096	265	574	3122	5333	889	132	258	76	4819		
	**Precision (%)**	92.7	51.3	22	90.7	0.3									
