# Supplementary material for: Machine learning with taxonomic family delimitation aids in the classification of ephemeral beaked whale events in passive acoustic monitoring
Source: PLoS One. 2024 Jun 4;19(6):e0304744. doi: 10.1371/journal.pone.0304744 (PMC11149863; doi:10.1371/journal.pone.0304744)
Supplement: S1 Fig — (PDF) [file pone.0304744.s006.pdf]

Supplementary Material

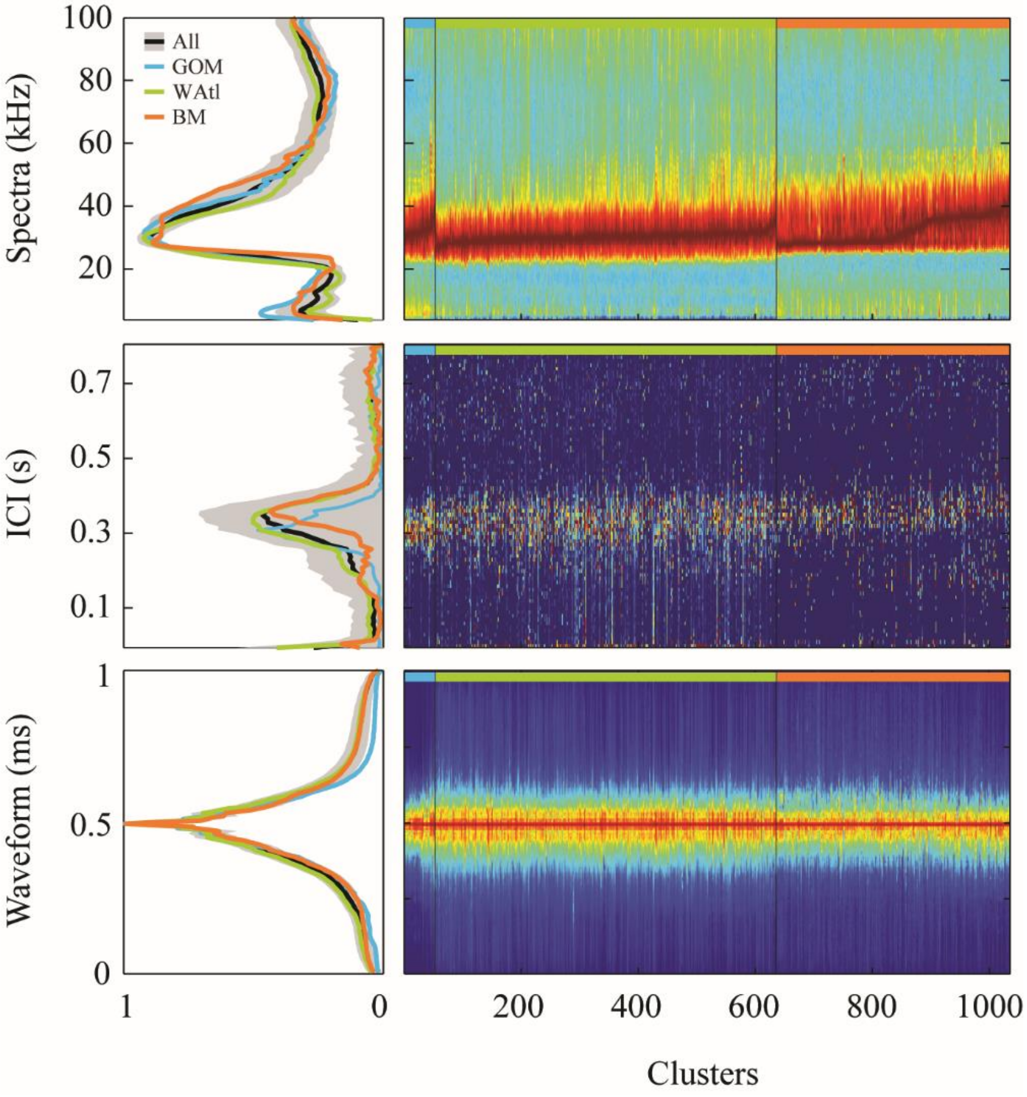

Figure S1. Blainville's beaked whale signal class organized by region formed using unsupervised clustering at a 5-min bin level based on spectra, inter-click interval (ICI), and waveform envelope. Top panels depict the mean and standard deviation of spectra among all clusters (top left) and concatenated mean cluster spectra (top right); the middle panels depict the mean and standard deviation of ICI distributions among all clusters (middle left) and concatenated cluster ICI distributions (middle right); and the bottom panels depict the mean and standard deviation of waveform envelops among all clusters (bottom left) and concatenated mean cluster waveform envelope (bottom right). Concatenated clusters have been sorted by region and peak frequency. Color map represents normalized amplitudes on a scale from 0 (dark blue) to 1 (dark red).
