# Supplementary material for: Machine learning with taxonomic family delimitation aids in the classification of ephemeral beaked whale events in passive acoustic monitoring
Source: PLoS One. 2024 Jun 4;19(6):e0304744. doi: 10.1371/journal.pone.0304744 (PMC11149863; doi:10.1371/journal.pone.0304744)
Supplement: S2 Fig — (PDF) [file pone.0304744.s007.pdf]

Supplementary Material

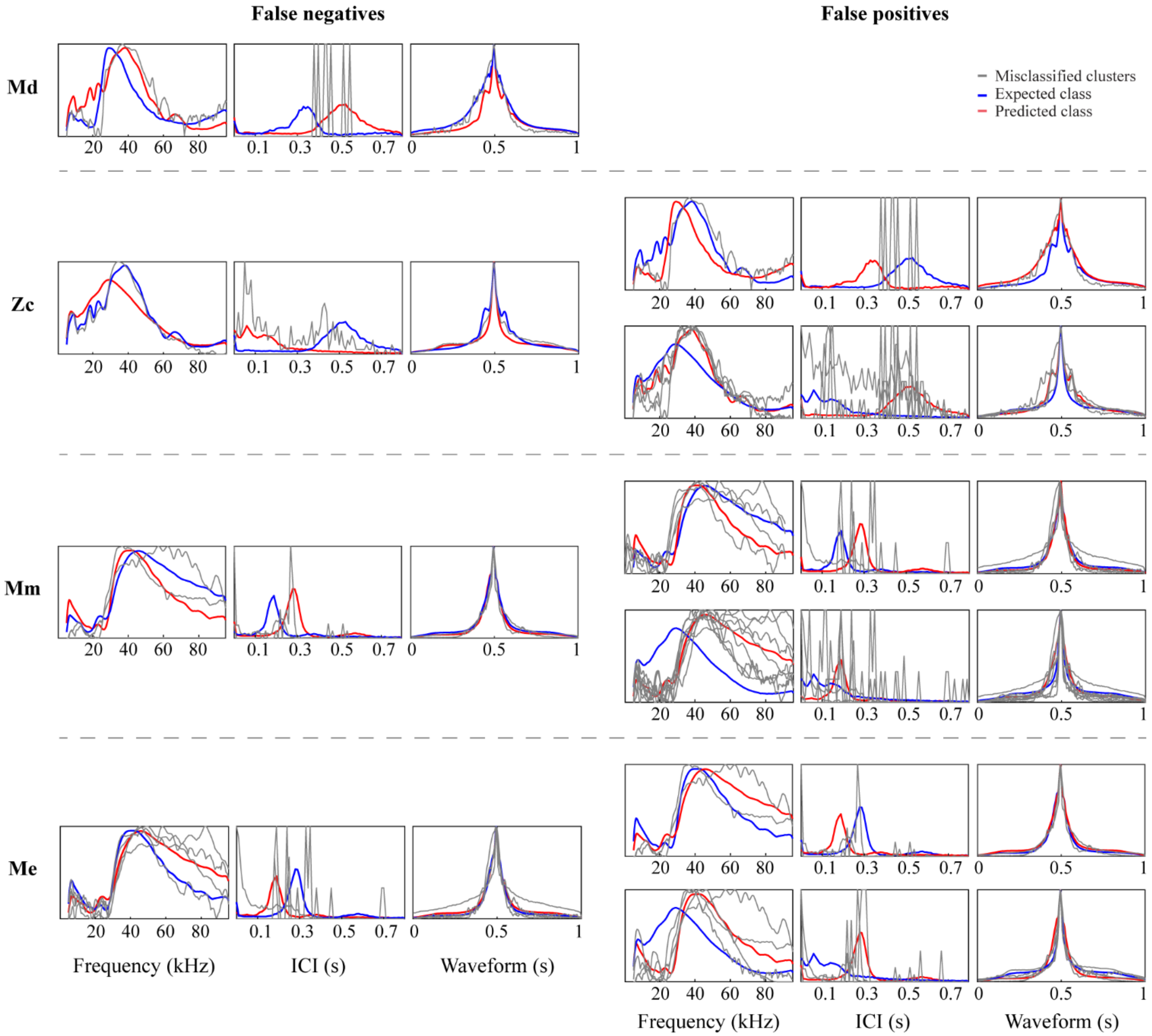

Figure S2. Clusters misclassified by the deep neural network in the balanced test set. Only beaked whale classes which contained misclassifications are shown. Gray lines depict the distribution of each cluster misclassified, and as reference the blue line depicts the average distribution of the expected class, and the red line of the predicted class. Refer to **Table 1** for abbreviation IDs.
