# Supplementary material for: Machine learning with taxonomic family delimitation aids in the classification of ephemeral beaked whale events in passive acoustic monitoring
Source: PLoS One. 2024 Jun 4;19(6):e0304744. doi: 10.1371/journal.pone.0304744 (PMC11149863; doi:10.1371/journal.pone.0304744)
Supplement: S1 File — (DOCX) [file pone.0304744.s008.docx]

**Machine learning with taxonomic family delimitation aids in the classification of ephemeral beaked whale events in passive acoustic monitoring**

**Supplementary Material**

**Table S1. Deployment summary for all regions and sites with HARP recordings.** Number of deployments per site, location, bottom depth, start and end date of effort, total days with recording, hydrophone crossover frequency between low- and high-frequency band sensor elements, data use for training, and case study (CS). Region abbreviations: Western North Atlantic – WAT and Gulf of Mexico – GOM.

| **Region** | **Site** | **Deployment** | **Latitude (°N)** | **Longitude (°W)** | **Depth (m)** | **Start date (UTC)** | **End date (UTC)** | **Recording days** | **Crossover freq. (kHz)** | **Data usage** |
| --- | --- | --- | --- | --- | --- | --- | --- | --- | --- | --- |
| **WAT** | HZ – Heezen Canyon | 1 | 41-03.715 | 66-21.092 | 845 | 06/27/2015 | 03/25/2016 | 271 | 25 | Training |
|  |  | 2 | 41-03.710 | 66-21.095 | 845 | 07/01/2016 | 08/31/2016 | 62 | -- | CS |
|  | OC – Oceanographer Canyon | 1 | 40-15.799 | 67-59.174 | 450 | 07/01/2016 | 08/31/2016 | 62 | -- | CS |
|  | BR – Bear Seamount | 1 | 40-01.967 | 67-59.301 | 2085 | 07/25/2018 | 08/17/2018 | 23 | -- | Training |
|  | NC – Nantucket Canyon | 1 | 39-49.949 | 69-58.928 | 977 | 04/27/2015 | 09/18/2015 | 145 | 25 | Training |
|  |  | 2 | 39-49.943 | 69-58.926 | 977 | 07/01/2016 | 08/31/2016 | 62 | -- | CS |
|  | BC – Babylon Canyon | 1 | 39-11.463 | 72-13.722 | 1000 | 07/01/2016 | 08/31/2016 | 62 | -- | CS |
|  | WC – Wilmington Canyon | 1 | 38-22.449 | 73-22.241 | 1000 | 07/01/2016 | 08/31/2016 | 62 | -- | CS |
|  | NFC – Norfolk Canyon | 2 | 37-09.991 | 74-27.996 | 968 | 04/30/2016 | 06/28/2017 | 424 | -- | Training |
|  |  | 3 | 37-10.044 | 74-27.980 | 950 | 06/30/2017 | 06/02/2018 | 337 | 2 | Training |
|  | HAT – Hatteras | 1 | 35-35.011 | 74-44.584 | 1200 | 07/01/2016 | 08/31/2016 | 62 | -- | Training |
|  | ONB – Onslow Bay | 1 | 33-46.676 | 75-55.585 | 952 | 08/19/2011 | 12/01/2011 | 105 | 2 | Training |
|  |  | 2 | 33-47.200 | 75-55.750 | 914 | 07/14/2012 | 10/02/2012 | 81 | 2 | Training |
|  | GS – Gulf Stream | 1 | 33-39.938 | 76-00.083 | 953 | 07/01/2016 | 08/31/2016 | 62 | -- | CS |
|  | BP – Blake Plateau | 1 | 32-06.362 | 77-05.659 | 945 | 07/01/2016 | 08/31/2016 | 62 | -- | CS |
|  | BM – Bermuda | 1 | 31-55.575 | 65-12.900 | 713 | 06/10/2013 | 03/11/2014 | 274 | 25 | Training |
|  |  | 2 | 31-55.415 | 65-12.113 | 732 | 03/13/2014 | 07/21/2014 | 131 | 25 | Training |
|  |  | 3 | 31-55.415 | 65-12.113 | 732 | 12/17/2014 | 10/02/2015 | 289 | 25 | Training |
|  | BS – Blake Spur | 1 | 30-35.027 | 77-23.443 | 1005 | 04/27/2016 | 06/26/2017 | 425 | -- | Training |
|  | JAX – Jacksonville | 1 | 30-09.036 | 79-46.203 | 800 | 08/23/2014 | 05/29/2015 | 279 | 10 | Training |
|  |  | 2 | 30-09.135 | 79-46.236 | 740 | 06/27/2018 | 06/15/2019 | 354 | -- | Training |
| **GOM** | MC – Mississippi Canyon | 1 | 28-50.797 | 88-27.991 | 980 | 09/22/2011 | 02/21/2012 | 152 | 2 | Training |
|  |  | 2 | 28-50.853 | 88-28.041 | 980 | 02/28/2012 | 12/11/2012 | 288 | 2 | Training |
|  | GC – Green Canyon | 1 | 27-33.470 | 91-10.010 | 1115 | 07/15/2010 | 10/11/2010 | 88 | 2 | Training |
|  |  | 2 | 27-33.466 | 91-10.014 | 1160 | 11/08/2010 | 02/02/2011 | 86 | 2 | Training |
|  |  | 3 | 27-33.424 | 91-10.073 | 1100 | 03/23/2011 | 08/08/2011 | 138 | 2 | Training |
|  |  | 4 | 27-33.426 | 91-10.060 | 1100 | 09/23/2011 | 02/17/2012 | 118 | 2 | Training |
|  |  | 5 | 27-33.440 | 91-10.562 | 1100 | 02/28/2012 | 12/12/2012 | 289 | 2 | Training |
|  | DT – Dry Tortugas | 1 | 25-31.911 | 84-38.251 | 1320 | 08/09/2010 | 10/26/2010 | 79 | 2 | Training |
|  |  | 2 | 25-31.911 | 84-38.251 | 1320 | 03/04/2011 | 06/24/2011 | 111 | 2 | Training |
|  |  | 3 | 25-32.360 | 84-37.743 | 1210 | 06/22/2016 | 07/18/2017 | 392 | -- | Training |
|  | HH – Howell Hook | 1 | 25-01.702 | 84-23.769 | 1050 | 05/27/2012 | 12/06/2012 | 194 | 2 | Training |

**Table S2. Detection summary for the manual and targeted species classification pipeline from the case study dataset.** Total number of 5-minute bins with species presence across all sites between July and August 2016. The targeted species classification pipeline implemented a hard negative filter.

|  | | **HZ** | **OC** | **NC** | **BC** | **WC** | **GS** | **BP** | **All Sites** |
| --- | --- | --- | --- | --- | --- | --- | --- | --- | --- |
| **Manual classification:** | |  |  |  |  |  |  |  |  |
|  | BWG | 0 | 0 | 0 | 0 | 0 | 0 | 0 | 0 |
|  | Mb | 297 | 45 | 25 | 59 | 421 | 0 | 0 | 847 |
|  | Md | 0 | 0 | 0 | 0 | 0 | 0 | 6 | 6 |
|  | Me | 0 | 0 | 0 | 1 | 0 | 1033 | 2157 | 3191 |
|  | Mm | 0 | 0 | 94 | 90 | 162 | 0 | 0 | 346 |
|  | Zc | 786 | 3 | 24 | 141 | 374 | 6 | 0 | 1334 |
|  |  |  |  |  |  |  |  |  |  |
| **Neural network classification:** | |  |  |  |  |  |  |  |  |
|  | BWG | 113 | 350 | 1893 | 119 | 182 | 59 | 70 | 2786 |
|  | Mb | 280 | 36 | 13 | 59 | 404 | 0 | 6 | 798 |
|  | Md | 82 | 214 | 291 | 68 | 55 | 5 | 9 | 724 |
|  | Me | 48 | 32 | 69 | 40 | 33 | 864 | 1738 | 2824 |
|  | Mm | 45 | 93 | 326 | 153 | 179 | 168 | 675 | 1639 |
|  | Zc | 828 | 100 | 157 | 200 | 489 | 26 | 48 | 1848 |

**Table S3. Confusion matrix for the targeted species classification pipeline with a hard negative filter on site Heezen Canyon (HZ).** Values indicate the total number of 5-minute bins classified. Refer to **Table 1** for abbreviation IDs. Bins with no clear assignment are indicated with the class abbreviation in italic.

| **Site HZ** | | **Predicted Class** | | | | | | | | | | | |  |  |
| --- | --- | --- | --- | --- | --- | --- | --- | --- | --- | --- | --- | --- | --- | --- | --- |
|  |  | Mb | Zc | BWG | Md | Me | Mm | De spp | Gg | Ko spp | Pm-boat | ES ping | *No label* | **N missed** | **Recall  (%)** |
| **True Class** | Mb | **274** |  |  | 2 |  | 4 | 8 |  | 2 | 1 |  | 8 | 25 | 91.6 |
|  | Zc | 1 | **713** | 6 | 3 | 9 | 10 | 31 | 10 |  |  |  | 5 | 75 | 90.5 |
|  | *Mm–Me* |  |  |  |  |  | 4 |  |  |  |  |  |  | 4 |  |
|  | *No label* | 5 | 115 | 107 | 77 | 39 | 29 | 597 | 441 | 6 | 4 | 4 | 698 | 2122 |  |
| **N false alarm** | | 6 | 115 | 113 | 82 | 48 | 47 | 636 | 451 | 8 | 5 | 4 | 711 |  |  |
| **Precision (%)** | | 97.9 | 86.1 |  |  |  |  |  |  |  |  |  |  |  |  |

**Table S4. Confusion matrix for the targeted species classification pipeline with a hard negative filter on site Oceanographic Canyon (OC).** Values indicate the total number of 5-minute bins classified. Refer to **Table 1** for abbreviation IDs. Bins with no clear assignment are indicated with the class abbreviation in italic.

| **Site OC** | | **Predicted Class** | | | | | | | | | | | |  |  |
| --- | --- | --- | --- | --- | --- | --- | --- | --- | --- | --- | --- | --- | --- | --- | --- |
|  |  | Mb | Zc | BWG | Md | Me | Mm | De spp | Gg | Ko spp | Pm-boat | ES ping | *No label* | **N missed** | **Recall  (%)** |
| **True Class** | Mb | **35** |  | 2 |  |  | 3 | 3 | 1 |  |  |  | 1 | 10 | 77.8 |
|  | Zc |  | **1** |  |  | 1 |  | 1 |  |  |  |  |  | 2 | 33.3 |
|  | *Mm–Me* |  |  |  |  | 1 | 1 |  |  |  |  |  |  | 2 |  |
|  | *likely Md* |  |  |  |  |  |  | 1 |  |  |  |  |  | 1 |  |
|  | *No label* | 1 | 99 | 348 | 214 | 30 | 89 | 1133 | 495 | 8 | 10 | 7 | 1331 | 3765 |  |
| **N false alarm** | | 1 | 99 | 350 | 214 | 32 | 93 | 1138 | 496 | 8 | 10 | 7 | 1332 |  |  |
| **Precision (%)** | | 97.2 | 1.0 |  |  |  |  |  |  |  |  |  |  |  |  |

**Table S5. Confusion matrix for the targeted species classification pipeline with a hard negative filter on site Nantucket Canyon (NC).** Values indicate the total number of 5-minute bins classified. Refer to **Table 1** for abbreviation IDs. Bins with no clear assignment are indicated with the class abbreviation in italic.

| **Site NC** | | **Predicted Class** | | | | | | | | | | | |  |  |
| --- | --- | --- | --- | --- | --- | --- | --- | --- | --- | --- | --- | --- | --- | --- | --- |
|  |  | Mm | Zc | Mb | BWG | Md | Me | De spp | Gg | Ko spp | Pm-boat | ES ping | *No label* | **N missed** | **Recall  (%)** |
| **True Class** | Mm | **88** | 1 |  | 3 |  |  | 1 |  | 1 |  |  | 1 | 7 | 92.6 |
|  | Zc | 2 | **20** |  | 3 | 1 |  |  |  |  |  |  |  | 6 | 76.9 |
|  | Mb | 1 |  | **9** | 2 |  |  | 2 |  |  | 1 |  | 10 | 16 | 36.0 |
|  | *Mm–Me* | 11 | 1 |  | 2 |  |  | 1 |  |  |  |  |  | 15 |  |
|  | *likely Md* |  |  |  |  |  |  |  |  | 1 |  |  |  | 1 |  |
|  | *No label* | 225 | 135 | 4 | 1884 | 290 | 69 | 843 | 630 | 50 | 166 | 37 | 998 | 5331 |  |
| **N false alarm** | | 239 | 137 | 4 | 1894 | 291 | 69 | 847 | 630 | 52 | 167 | 37 | 1009 |  |  |
| **Precision (%)** | | 26.9 | 12.7 | 69.2 |  |  |  |  |  |  |  |  |  |  |  |

**Table S6. Confusion matrix for the targeted species classification pipeline with a hard negative filter on site Babylon Canyon (BC).** Values indicate the total number of 5-minute bins classified. Refer to **Table 1** for abbreviation IDs. Bins with no clear assignment are indicated with the class abbreviation in italic.

| **Site BC** | | **Predicted Class** | | | | | | | | | | | |  |  |
| --- | --- | --- | --- | --- | --- | --- | --- | --- | --- | --- | --- | --- | --- | --- | --- |
|  |  | Me | Mb | Mm | Zc | BWG | Md | De spp | Gg | Ko spp | Pm-boat | ES ping | *No label* | **N missed** | **Recall  (%)** |
| **True Class** | Me | **1** |  |  |  |  |  |  |  |  |  |  |  | 0 | 100.0 |
|  | Mb | 1 | **55** |  |  |  |  | 2 |  | 1 |  |  |  | 4 | 93.2 |
|  | Mm | 2 |  | **84** | 3 | 2 |  | 4 |  |  |  |  | 1 | 12 | 87.5 |
|  | Zc |  |  | 5 | **125** | 7 | 2 | 2 | 3 |  |  |  | 1 | 20 | 86.2 |
|  | *Mm–Me* | 2 |  | 5 |  |  |  | 1 |  |  |  |  |  | 8 |  |
|  | *No label* | 34 | 4 | 61 | 74 | 111 | 67 | 468 | 364 | 12 | 4 | 1 | 390 | 1590 |  |
| **N false alarm** | | 39 | 4 | 71 | 77 | 120 | 69 | 477 | 367 | 13 | 4 | 1 | 392 |  |  |
| **Precision (%)** | | 2.5 | 93.2 | 54.2 | 61.9 |  |  |  |  |  |  |  |  |  |  |

**Table S7. Confusion matrix for the targeted species classification pipeline with a hard negative filter on site Wilmington Canyon (WC).** Values indicate the total number of 5-minute bins classified. Refer to **Table 1** for abbreviation IDs. Bins with no clear assignment are indicated with the class abbreviation in italic.

| **Site WC** | | **Predicted Class** | | | | | | | | | | | |  |  |
| --- | --- | --- | --- | --- | --- | --- | --- | --- | --- | --- | --- | --- | --- | --- | --- |
|  |  | Mb | Zc | Mm | BWG | Md | Me | De spp | Gg | Ko spp | Pm-boat | ES ping | *No label* | **N missed** | **Recall  (%)** |
| **True Class** | Mb | **393** | 1 | 6 | 2 |  |  | 12 |  |  |  |  | 11 | 32 | 92.5 |
|  | Zc |  | **326** | 2 | 4 |  | 2 | 27 | 7 | 1 | 1 | 3 | 5 | 52 | 86.2 |
|  | Mm | 4 | 2 | **143** |  |  | 9 | 4 | 1 | 2 |  |  | 3 | 25 | 85.1 |
|  | *Mm–Me* |  |  | 9 |  |  | 2 |  |  |  |  |  |  | 11 |  |
|  | *No label* | 9 | 160 | 22 | 176 | 55 | 21 | 1147 | 161 | 4 | 33 | 47 | 891 | 2726 |  |
| **N false alarm** | | 13 | 163 | 39 | 182 | 55 | 34 | 1190 | 169 | 7 | 34 | 50 | 910 |  |  |
| **Precision (%)** | | 96.8 | 66.7 | 78.6 |  |  |  |  |  |  |  |  |  |  |  |

**Table S8. Confusion matrix for the targeted species classification pipeline with a hard negative filter on site Gulf Stream (GS).** Values indicate the total number of 5-minute bins classified. Refer to **Table 1** for abbreviation IDs. Bins with no clear assignment are indicated with the class abbreviation in italic.

| **Site GS** | | **Predicted Class** | | | | | | | | | | | |  |  |
| --- | --- | --- | --- | --- | --- | --- | --- | --- | --- | --- | --- | --- | --- | --- | --- |
|  |  | Me | Zc | BWG | Mb | Md | Mm | De spp | Gg | Ko spp | Pm-boat | ES ping | *No label* | **N missed** | **Recall  (%)** |
| **True Class** | Me | **848** | 11 | 19 |  |  | 150 | 9 |  | 2 |  |  |  | 191 | 81.6 |
|  | Zc | 1 | **3** |  |  |  | 1 |  |  |  |  |  | 1 | 3 | 50.0 |
|  | *Mm – Me* |  |  | 1 |  |  | 2 |  |  |  |  |  |  | 3 |  |
|  | *likely Md* |  |  | 1 |  |  |  |  |  |  |  |  |  | 1 |  |
|  | *likely Me* |  |  | 1 |  |  |  |  |  |  |  |  |  | 1 |  |
|  | *No label* | 15 | 12 | 38 |  | 5 | 20 | 60 | 25 | 15 | 5 | 1 | 54 | 250 |  |
| **N false alarm** | | 16 | 23 | 60 | 0 | 5 | 173 | 69 | 25 | 17 | 5 | 1 | 55 |  |  |
| **Precision (%)** | | 98.1 | 11.5 |  |  |  |  |  |  |  |  |  |  |  |  |

**Table S9. Confusion matrix for the targeted species classification pipeline with a hard negative filter on site Blake Plateau (BP).** Values indicate the total number of 5-minute bins classified. Refer to **Table 1** for abbreviation IDs. Bins with no clear assignment are indicated with the class abbreviation in italic.

| **Site BP** | | **Predicted Class** | | | | | | | | | | | |  |  |
| --- | --- | --- | --- | --- | --- | --- | --- | --- | --- | --- | --- | --- | --- | --- | --- |
|  |  | Me | Md | BWG | Mb | Mm | Zc | De spp | Gg | Ko spp | Pm-boat | ES ping | *No label* | **N missed** | **Recall  (%)** |
| **True Class** | Me | **1714** | 4 | 63 | 3 | 336 | 40 | 23 | 3 | 5 |  | 1 |  | 478 | 78.2 |
|  | Md | 1 | **2** | 1 |  |  |  | 3 |  |  |  |  |  | 5 | 28.6 |
|  | *Md – Me* | 1 |  |  |  |  |  |  |  |  |  |  |  | 1 |  |
|  | *No label* | 23 | 5 | 7 | 5 | 363 | 9 |  | 79 | 70 |  |  | 410 | 971 |  |
| **N false alarm** | | 25 | 9 | 71 | 8 | 699 | 49 | 26 | 82 | 75 | 0 | 1 | 410 |  |  |
| **Precision (%)** | | 98.6 | 100 |  |  |  |  |  |  |  |  |  |  |  |  |

**Table S10. Confusion matrix the targeted species classification pipeline with a hard negative filter on site Wilmington Canyon (WC).** Values indicate the total number of 5-minute bins classified. Refer to **Table 1** for abbreviation IDs. Bins with no clear assignment are indicated with the class abbreviation in italic.

| **Site WC** | | **Predicted Class** | | | | | | | | | | | |  |  |
| --- | --- | --- | --- | --- | --- | --- | --- | --- | --- | --- | --- | --- | --- | --- | --- |
|  |  | Mb | Zc | Mm | BWG | Md | Me | De spp | Gg | Ko spp | Pm-boat | ES ping | *No label* | **N missed** | **Recall  (%)** |
| **True Class** | Mb | **391** | 1 | 6 | 2 |  |  | 12 |  |  |  |  | 11 | 32 | 92.5 |
|  | Zc |  | **326** | 2 | 4 |  | 2 | 27 | 7 | 1 | 1 | 3 | 5 | 52 | 86.2 |
|  | Mm | 4 | 2 | **143** |  |  | 9 | 4 | 1 | 2 |  |  | 3 | 25 | 85.1 |
|  | *Mm–Me* |  |  | 9 |  |  | 2 |  |  |  |  |  |  | 11 |  |
|  | *No label* | 9 | 160 | 22 | 176 | 55 | 21 | 1147 | 161 | 4 | 33 | 47 | 891 | 2726 |  |
| **N false alarm** | | 13 | 163 | 39 | 182 | 55 | 34 | 1190 | 169 | 7 | 34 | 50 | 910 |  |  |
| **Precision (%)** | | 96.1 | 66.7 | 78.6 |  |  |  |  |  |  |  |  |  |  |  |

**Table S11. Confusion matrix for the targeted species classification pipeline with a hard negative filter on the case study dataset with training sample size 1000 and added noise to increase variability.** Values indicate the total number of 5-minute bins classified. Refer to **Table 1** for abbreviation IDs. Bins with no clear assignment are indicated with the class abbreviation in italic.

|  | |  |  |  |  |  |  |  |  |  |  |  |  |  |  |
| --- | --- | --- | --- | --- | --- | --- | --- | --- | --- | --- | --- | --- | --- | --- | --- |
|  | | Mb | Zc | Mm | Me | Md | BWG | De spp | Gg | Ko spp | Pm-boat | ES ping | *No label* | **N missed** | **Recall  (%)** |
| **True Class** | Mb | **776** | 3 | 5 |  | 1 | 14 | 20 |  | 2 | 1 |  | 30 | 76 | 91.1 |
|  | Zc | 1 | **1206** | 10 | 19 | 1 | 20 | 65 | 8 | 1 |  | 2 | 12 | 139 | 89.7 |
|  | Mm | 4 | 10 | **309** | 12 |  | 10 | 6 |  | 2 |  |  | 5 | 49 | 86.3 |
|  | Me | 9 | 66 | 411 | **2562** | 3 | 131 | 37 | 4 | 7 |  |  |  | 668 | 79.3 |
|  | Md |  |  |  | 1 | **2** | 1 | 3 |  |  |  |  |  | 5 | 28.6 |
|  | *Mm – Me* |  |  | 23 | 9 |  | 5 | 5 | 1 |  |  |  |  | 43 |  |
|  | *Md – Me* |  |  |  | 1 |  |  |  |  |  |  |  |  | 1 |  |
|  | *likely Me* |  |  |  |  |  |  | 1 |  |  |  |  |  | 1 |  |
|  | *likely Md* |  |  |  |  |  |  | 3 |  |  |  |  |  | 3 |  |
|  | *No label* | 47 | 1067 | 647 | 222 | 569 | 2941 | 5193 | 876 | 120 | 257 | 74 | 4772 | 16785 |  |
| **N false alarm** | | 61 | 1146 | 1096 | 265 | 574 | 3122 | 5333 | 889 | 132 | 258 | 76 | 4819 |  |  |
| **Precision (%)** | | 92.7 | 51.3 | 22 | 90.7 | 0.3 |  |  |  |  |  |  |  |  |  |

**Table S12. Confusion matrix for the targeted species classification pipeline with a hard negative filter on the case study dataset with training sample size 5000 and added noise to increase variability.** Values indicate the total number of 5-minute bins classified. Refer to **Table 1** for abbreviation IDs. Bins with no clear assignment are indicated with the class abbreviation in italic.

|  | |  |  |  |  |  |  |  |  |  |  |  |  |  |  |
| --- | --- | --- | --- | --- | --- | --- | --- | --- | --- | --- | --- | --- | --- | --- | --- |
|  | | Mm | Mb | Zc | Me | Md | BWG | De spp | Gg | Ko spp | Pm-boat | ES ping | *No label* | **N missed** | **Recall  (%)** |
| **True Class** | Mm | **311** | 4 | 3 | 10 |  | 15 | 10 |  | 1 |  |  | 5 | 48 | 86.6 |
|  | Mb | 35 | **711** | 1 | 4 | 1 | 18 | 51 |  | 1 | 1 |  | 30 | 142 | 83.4 |
|  | Zc | 6 | 1 | **1056** | 13 | 14 | 59 | 174 | 11 |  | 1 | 2 | 12 | 293 | 78.3 |
|  | Me | 438 |  | 13 | **2436** | 6 | 184 | 145 | 15 | 3 |  |  |  | 804 | 75.2 |
|  | Md |  |  |  | 1 | **2** | 1 | 3 |  |  |  |  |  | 5 | 28.6 |
|  | *Mm – Me* | 22 |  |  | 3 | 1 | 9 | 8 |  |  |  |  |  | 43 |  |
|  | *Md – Me* |  |  |  | 1 |  |  |  |  |  |  |  |  | 1 |  |
|  | *likely Me* |  |  |  |  |  | 1 |  |  |  |  |  |  | 1 |  |
|  | *likely Md* |  |  |  |  |  | 2 | 1 |  |  |  |  |  | 3 |  |
|  | *No label* | 418 | 8 | 297 | 144 | 491 | 3238 | 5913 | 1166 | 14 | 145 | 101 | 4772 | 16707 |  |
| **N false alarm** | | 919 | 13 | 314 | 176 | 513 | 3527 | 6305 | 1192 | 19 | 147 | 103 | 4819 |  |  |
| **Precision (%)** | | 25.3 | 98.2 | 77.1 | 93.3 | 0.4 |  |  |  |  |  |  |  |  |  |

**
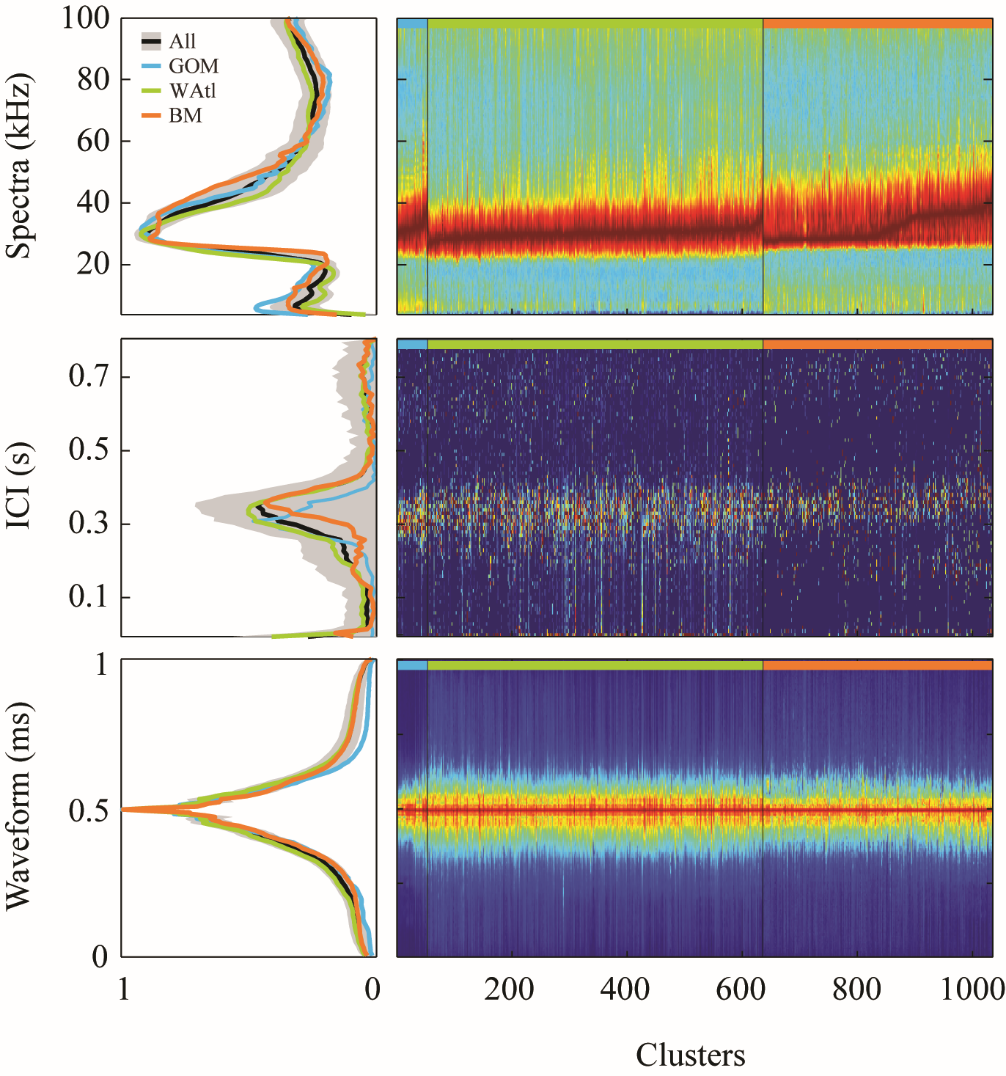
**

**Figure S1. Blainville’s beaked whale signal class organized by region formed using unsupervised clustering at a 5-min bin level based on spectra, inter-click interval (ICI), and waveform envelope.** Top panels depict the mean and standard deviation of spectra among all clusters (top left) and concatenated mean cluster spectra (top right); the middle panels depict the mean and standard deviation of ICI distributions among all clusters (middle left) and concatenated cluster ICI distributions (middle right); and the bottom panels depict the mean and standard deviation of waveform envelops among all clusters (bottom left) and concatenated mean cluster waveform envelope (bottom right). Concatenated clusters have been sorted by region and peak frequency. Color map represents normalized amplitudes on a scale from 0 (dark blue) to 1 (dark red).


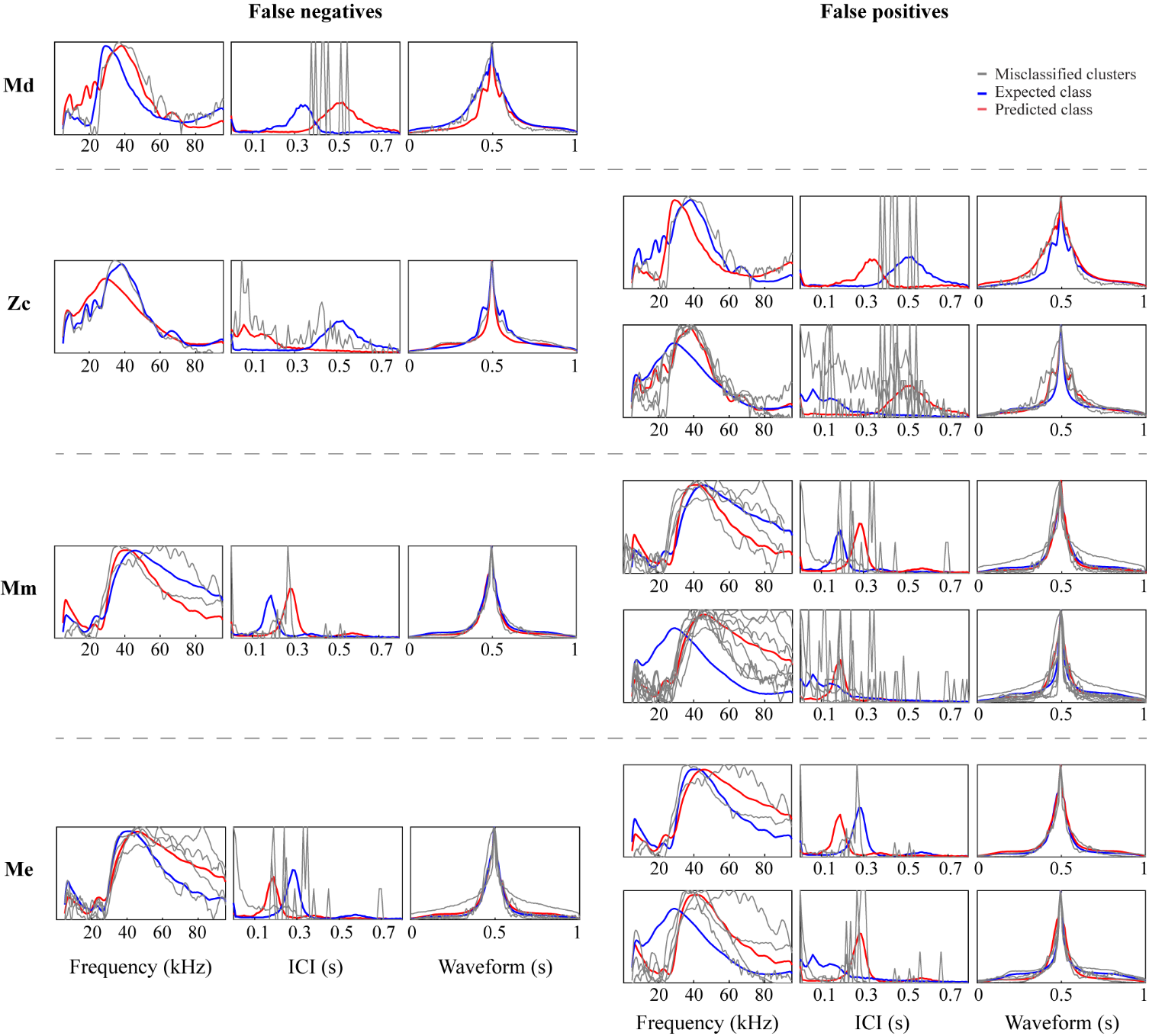


**Figure S2. Clusters misclassified by the deep neural network in the balanced test set.** Only beaked whale classes which contained misclassifications are shown. Gray lines depict the distribution of each cluster misclassified, and as reference the blue line depicts the average distribution of the expected class, and the red line of the predicted class. Refer to **Table 1** for abbreviation IDs.
